# Brain metastasis-associated cancer fibroblasts drive tumor progression and therapeutic resistance through IL26 and CX3CL1 signaling in non-small-cell lung cancer

**DOI:** 10.1186/s40164-025-00713-9

**Published:** 2025-09-30

**Authors:** S. M. Abdus Salam, Eshrat Jahan, Eun-Jung Ahn, Sung Sun Kim, Yeong Jin Kim, Sue Jee Park, Tae-Young Jung, In-Young Kim, Shin Jung, Roo Ji Lee, Jae-Hyuk Lee, Joon Haeng Rhee, Kyung Keun Kim, Min-Hee Yi, Kyung-Hwa Lee, Kyung-Sub Moon

**Affiliations:** 1 Department of Neurosurgery, Chonnam National University Hwasun Hospital and Medical School, 322 Seoyang-ro, Hwasun-eup, Hwasun-gun, Hwasun, 58128 Jeollanam-do South Korea; 2 Department of Pathology, Chonnam National University Hwasun Hospital and Medical School, 322 Seoyang-ro, Hwasun-eup, Hwasun-gun, Hwasun, 58128 Jeollanam-do South Korea; 3 Department of Rehabilitation, Chonnam National University Hwasun Hospital and Medical School, Hwasun, South Korea; 4 Medical Research Center (MRC) for Immunotherapy of Cancer, Chonnam National University Medical School, Hwasun, South Korea; 5 Department of Pharmacology, Chonnam National University Medical School, Hwasun, South Korea; 6 Department of Microbiology and Immunology, Chonnam National University Medical School, Hwasun, South Korea; 7 BioMedical Sciences Graduate Program (BMSGP), Chonnam National University, Hwasun, South Korea

**Keywords:** Brain neoplasms, Cytokines, Neoplasm metastasis, Cancer-associated fibroblast, Neoplasm drug resistance, Non-small-cell lung carcinoma, Tumor microenvironment

## Abstract

Brain metastases (BM) from non-small cell lung cancer (NSCLC) represent a significant clinical challenge, characterized by poor prognosis and treatment resistance. While cancer-associated fibroblasts (CAFs) are recognized as crucial components of the BM tumor microenvironment (TME), their mechanistic contributions to disease progression and therapeutic resistance remain poorly understood. In this study, we demonstrated that patient-derived BM-CAFs significantly enhanced NSCLC cell proliferation, migration, invasion and therapeutic resistance in vitro. Mechanistically, BM-CAFs promoted epithelial–mesenchymal transition (EMT) and cancer stem cell (CSC) phenotypes through upregulation of key transcription factors. In-vivo experiments showed that co-injection of NSCLC cells with BM-CAFs accelerated tumor growth and enhanced cisplatin resistance. Molecular analysis revealed these effects were mediated through distinct mechanisms whereby IL26 activated the JAK–STAT3 pathway, while CX3CL1 activated both JAK–STAT3 and AKT–mTOR pathways. Importantly, neutralizing antibodies against IL26 and CX3CL1 effectively suppressed their respective signaling pathways and reversed EMT and CSC characteristics. In summary, our findings establish the IL26 and CX3CL1 signaling as a critical mediator of BM-CAF-induced tumor progression and therapy resistance in NSCLC BM, suggesting a potential therapeutic strategy for this challenging disease.

**Supplementary Information:**

The online version contains supplementary material available at 10.1186/s40164-025-00713-9.

**To the editor**:

Brain metastasis (BM) occurs when extracranial systemic cancers spread to the brain, causing over 50% of brain cancer deaths with median survival of only 6 months. Lung cancer is the primary cause of BM, followed by breast cancer and melanoma [1]. BM occurs in 20–30% of non-small-cell lung cancer (NSCLC) patients and associated with a particularly poor prognosis [2]. While traditional treatments (radiation, surgery, radiosurgery) have been supplemented by immunotherapy, effectiveness remains limited by the complex tumor microenvironment (TME) [3]. Within the TME, cancer-associated fibroblasts (CAFs) promote invasion, metastasis, tumorigenesis, and therapy resistance through secreted factors that induce epithelial–mesenchymal transition (EMT) and cancer stem cell (CSC) properties in cancer cells [4, 5]. Although recent in silico analyses have characterized BM-CAFs [6] and studies have explored CAFs’ tumor-promoting effects in glioblastoma [7], their specific role in NSCLC BM remains unexplored and requires comprehensive in vitro and in vivo investigation.

Following our established protocol [8], we isolated BM-CAFs with diverse morphologies from brain tumor tissues and uniform spindle-shaped normal fibroblasts (NFs) from scalp tissues of NSCLC BM patients (Fig. 1A & Supplementary Table 1). Western blot analysis and immunofluorescence (IF) staining confirmed elevated Vimentin, α-SMA and PDGFR-β in BM-CAFs, which lacked epithelial and endothelial markers (Fig. 1B & Fig. S1). In parallel, mouse lung (ML)-NFs underwent phenotypic transformation into ML-CAFs when co-cultured with Lewis lung carcinoma (LLC1) cells, as evidenced by increased expression of α-SMA and PDGFR-β (Fig. S2). RNA sequencing (RNAseq) of patient-derived BM-CAFs and NFs (Supplementary Table 2) revealed distinct transcriptional profiles through differentially expressed gene (DEG) analysis, including unsupervised clustering (Fig. 1C), principal component analysis (PCA) (Fig. 1D), and volcano plot highlighting significantly upregulated genes in BM-CAFs (Fig. 1E). Gene ontology analysis identified BM-CAF-associated pathway enrichment across several biological functions (Fig. 1F).

**Fig. 1:**
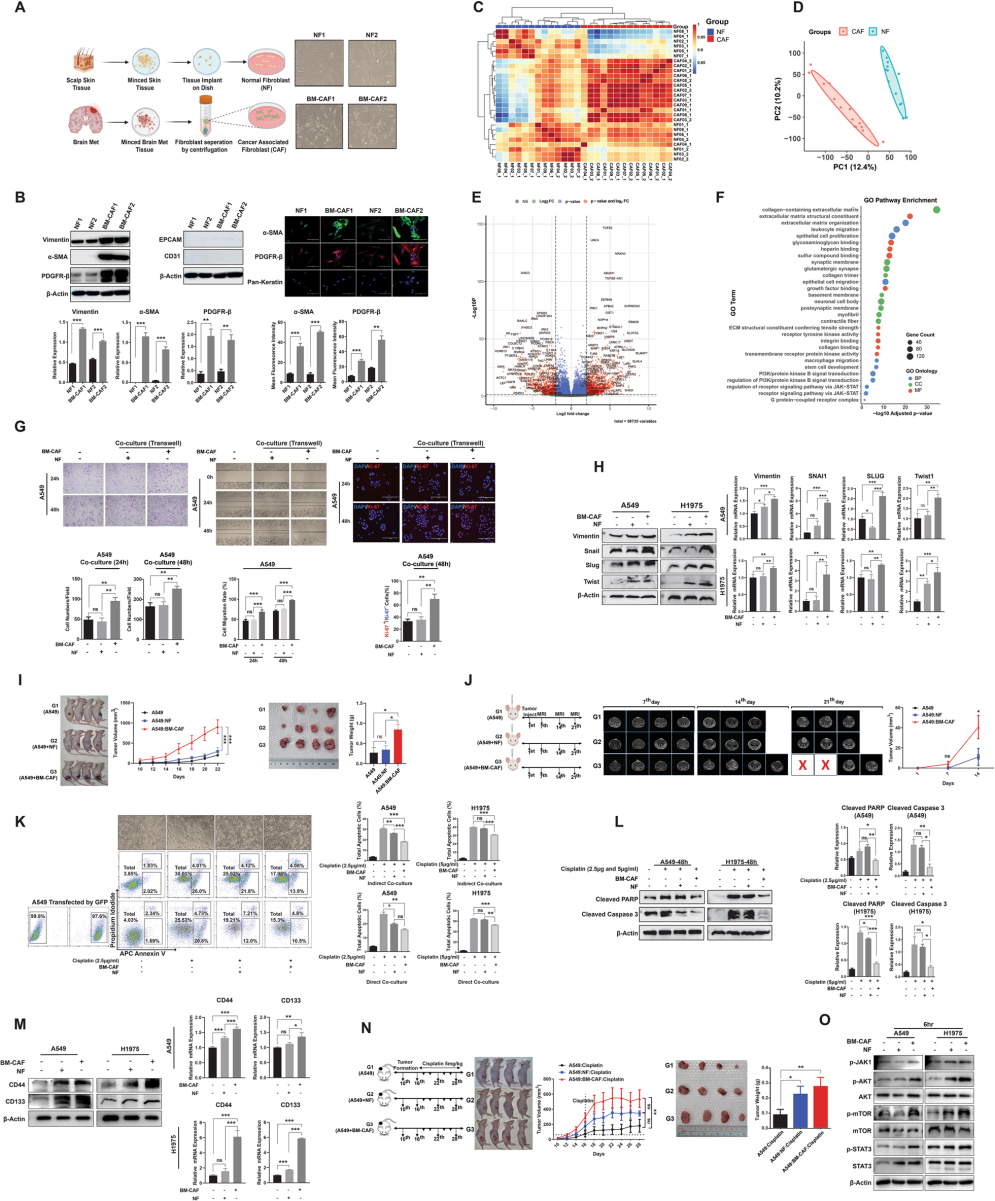
Biological role of patient-derived BM-CAF on NSCLC in vitro and in vivo. **A** Primary cultures of NFs and BM-CAFs from NSCLC BM patient specimens with phase-contrast images showing their morphology. **B** Western blot analysis showing higher expression of Vimentin, α-SMA, and PDGFR-β in BM-CAFs versus NFs, with undetectable EPCAM and CD31; proteins normalized to β-Actin. Immunofluorescence confirming elevated expression of α-SMA and PDGFR-β in BM-CAFs. **C** RNAseq and DEG analysis revealing distinct profiles between NFs and BM-CAFs via hierarchical clustering. **D** PCA showed PC1 and PC2 accounting for 12.4% and 10.2% of variance, distinguishing NF and BM-CAF transcriptomes. **E** Volcano plot showing significantly altered DEGs. **F** Gene ontology enrichment analysis identifying key pathways associated with BM-CAF upregulated genes. **G** BM-CAFs enhanced invasion, migration, and proliferation of A549 at 24 h and 48 h. **H** Western blot and qRT-PCR showing BM-CAF-induced upregulation of EMT markers in NSCLC cells. **I** In a subcutaneous xenograft model, co-injection of BM-CAFs with A549 cells (G3) resulted in larger tumor volume and weight compared to A549 alone (G1) or A549 with NFs (G2). **J** Orthotopic brain tumor model showing earlier and faster tumor growth with BM-CAF co-injection, confirmed by MRI. **K** Cisplatin assays showing enhanced viability and reduced apoptosis in A549 cells co-cultured with BM-CAFs. **L** Decreased cleaved PARP and cleaved caspase-3 expression in A549 and H1975 cells co-cultured with BM-CAFs. **M** BM-CAFs upregulating CSC markers CD44 and CD133 at protein and mRNA levels. **N** Larger tumors in the BM-CAF co-injection group after cisplatin treatment. **O** BM-CAF-induced activation of p-JAK1, p-AKT, p-mTOR, and p-STAT3 in NSCLC cells after 6 h co-culture. Results are presented as means ± standard error of the mean (SEM) and analyzed using one-way and two-way ANOVA followed by post hoc correction with Tukey’s and Bonferroni’s multiple comparison tests, with statistical significance indicated at *P < 0.05, **P < 0.01, and ***P < 0.001

In transwell assays, BM-CAFs significantly enhanced NSCLC cells invasion, migration, and proliferation (Fig. 1G & Fig. S3), which correlated with upregulation of EMT factors (Fig. 1H). BM-CAF co-injection in a subcutaneous xenograft model significantly enhanced tumor growth compared to both A549 cells alone and NFs co-injection groups, confirmed by final tumor weight measurements (Fig. 1I). Parallel studies with converted ML-CAFs showed similar trends (Fig. S4A). Further validation in an orthotopic brain tumor model using MRI revealed accelerated tumor formation and significantly larger tumor volumes in the BM-CAF co-injection group (Fig. 1J).

Using optimized cisplatin concentrations (Fig. S5), we found that BM-CAFs significantly reduced apoptotic populations of NSCLC cells in both indirect and direct coculture systems (Fig. 1K & Fig. S6). Western blot analysis confirmed reduced expression of apoptotic markers, including cleaved PARP and cleaved caspase-3, in NSCLC cells co-cultured with BM-CAFs or ML-CAFs (Fig. 1L & Fig. S7). BM-CAF also conferred modest resistance against radiation treatment (Fig. S8). The therapeutic resistance, particularly pronounced in chemotherapy response, correlated with increased expression of CSC factors in NSCLC cells co-cultured with BM-CAF (Fig. 1M), and this finding was further validated in vivo where BM-CAF co-injection resulted in enhanced expression of both EMT and CSC markers in mouse tumor tissues (Fig. S9). In cisplatin-treated xenograft models, tumors co-injected with BM-CAFs exhibited significantly increased tumor growth and weight (Fig. 1N), while parallel studies with converted ML-CAFs showed less pronounced but still notable protection against cisplatin (Fig. S4B). Mechanistically, BM-CAFs triggered rapid activation of JAK1, AKT, mTOR, and STAT3 phosphorylation in NSCLC cells (Fig. 1O).

KEGG pathway analysis of RNAseq data comparing patient-derived BM-CAFs and NFs revealed distinct pathway enrichment patterns in BM-CAFs (Fig. 2A). K-means clustering identified four distinct gene clusters, with Cluster D containing 296 upregulated genes (Fig. 2B & Supplementary Table 3). Within this cluster, the cytokine-cytokine receptor interaction pathway was particularly enriched, featuring 11 significantly upregulated genes in BM-CAFs (Fig. 2C). Among these cytokine genes, *CX3CL1* and *IL26* showed the most significant differential expression in BM-CAFs (*P* = 1e-04 and *P* = 4.8e-05, respectively; Fig. 2D & Fig. S10), which was validated by ELISA (*P* < 0.05 and *P* < 0.001, respectively; Fig. 2E). Parallel analysis of ML-NFs and ML-CAFs revealed similar cytokine expression patterns (Fig. 2F), with CX3CL1 consistently detected in the ML-CAF secretome, corroborating our findings from human BM-CAFs (Fig. 2G).

**Fig. 2:**
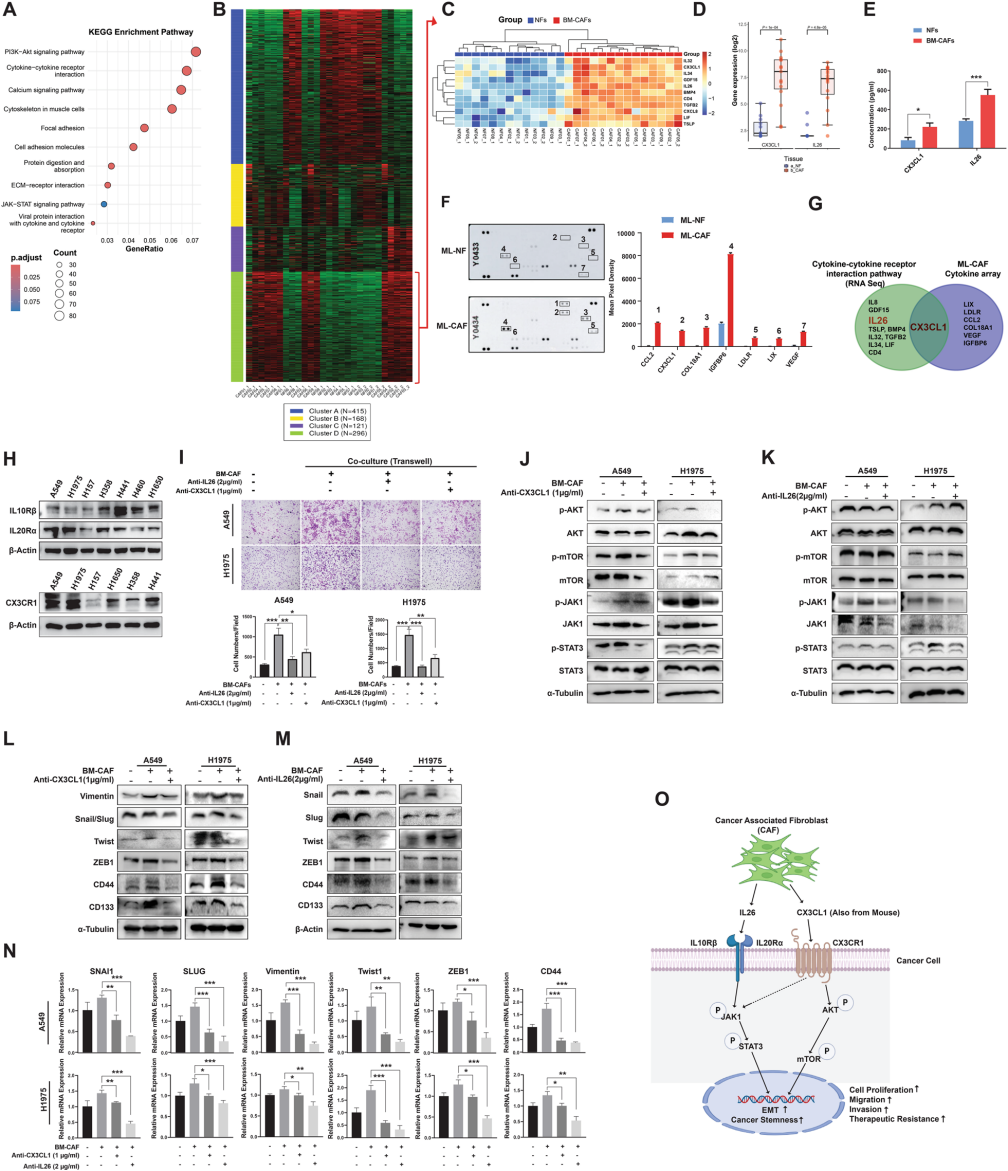
Cytokine axis of IL26 and CX3CL1 from BM-CAF on NSCLC cells. **A** KEGG enrichment analysis revealed key pathways associated with BM-CAF upregulated genes. **B** K-means clustering and KEGG pathway analysis identified four distinct DEG clusters, with Cluster D containing 296 upregulated genes. **C** Among these, 11 genes involved in cytokine-cytokine receptor interaction pathway were significantly upregulated within BM-CAFs, including *CX3CL1*,* IL32*,* CD4*,* TGFB2*,* IL26*,* BMP4*,* LIF*,* GDF15*,* TSLP*,* IL34*,* and CXCL8 (IL8)* on unsupervised hierarchical clustering heatmap. **D** Of these upregulated genes, *CX3CL1* and *IL26* demonstrated the highest expression levels with statistical significance (*P* = 1e-04 and *P* = 4.8e-05, respectively). **E** Enzyme-linked immunosorbent assay (ELISA) confirmed significantly increased secretion of CX3CL1 and IL26 in BM-CAFs compared to NFs. **F** & **G** Cytokine profiling of ML-NFs and ML-CAFs demonstrated elevated CX3CL1 levels in the ML-CAF secretome, corroborating the human RNA sequencing findings. **H** Expression analysis revealed the presence of IL26 (IL10Rβ/IL20Rα) and CX3CL1 (CX3CR1) receptors across all tested lung cancer cell lines. **I** Neutralizing IL26 or CX3CL1 significantly suppressed BM-CAF-induced invasion of cancer cells. **J**, **K** Neutralizing antibody experiments confirmed these signaling axes, with anti-CX3CL1 (1 µg/ml) treatment reducing CX3CL1 secretion and suppressing p-AKT, p-mTOR, and p-JAK1 activation, while anti-IL26 (2 µg/ml) treatment inhibited IL26-induced p-JAK1 and p-STAT3 activation. **L**–**N** Both anti-IL26 and anti-CX3CL1 treatments led to downregulation of EMT markers (Vimentin, Snail, Slug, Twist, ZEB1) and CSC markers (CD44, CD133) at protein and mRNA levels. **O** Schematic representation demonstrates that patient-derived BM-CAFs secrete IL26 and CX3CL1, which activate JAK–STAT3/AKT–mTOR signaling pathway in NSCLC cells, thereby promoting EMT and CSC characteristics, ultimately enhancing tumor progression and therapy resistance. Data are presented as means ± SEM with statistical significance indicated at *P < 0.05, **P < 0.01, and ***P < 0.001

Complementing these findings, various lung cancer cells expressed relevant receptors for IL26 (IL10Rβ/IL20Rα) and CX3CL1 (CX3CR1), confirming their responsiveness to BM-CAF-derived cytokines (Fig. 2H & Fig. S11). Mechanistically, the CX3CL1–CX3CR1 complex activates PI3K–AKT and JAK2–STAT3 pathways, contributing to apoptosis resistance and promoting CSC & EMT properties in human cancers [9–11]. Similarly, IL26 triggers multiple signaling cascades in various diseases, including JAK1/TYK2–STAT1/STAT3, PI3K/AKT, Raf/MEK/ERK, and NF-kB pathways, and drives cancer progression and therapeutic resistance [12–14]. The JAK1–STAT3 pathway directly maintains CSC and EMT factors, while the PI3K–AKT–mTOR axis supports CSC self-renewal, survival, and metabolic reprogramming [15]. To validate the functional significance of these BM-CAF-secreted cytokines, we used neutralizing antibodies and demonstrated that blockade of IL26 and CX3CL1 significantly reduced cancer cell invasion, proliferation, and chemoresistance (Fig. 2I & Figs. S12, 13). Anti-CX3CL1 neutralizing antibodies effectively reduced p-AKT, p-mTOR, and p-JAK1 (Fig. 2J), while anti-IL26 antibodies suppressed p-JAK1 and p-STAT3 (Fig. 2K). Importantly, neutralization of these cytokines resulted in decreased expression of both EMT and CSC factors (Fig. 2L–N), and similar results were confirmed using siRNA-mediated knockdown experiments (Fig. S14). However, in BM-CAF co-injection in vivo experiments using neutralizing antibodies against these cytokines, only anti-IL26 antibody treatment demonstrated significant tumor growth inhibition effects (Fig. S15). siRNA-mediated knockdown of IL26 and CX3CL1 led to a significant reduction in pSTAT3 in BM-CAFs (Fig. S16), but the upstream regulatory pathways and potential interlinking mechanisms between IL26 and CX3CL1 require further investigation. Immunohistochemical analysis of IL26 and CX3CL1 in NSCLC BM specimens (Fig. S17) revealed that high expression of both markers was associated with shorter recurrence-free survival, with CX3CL1 showing marginal statistical significance (*P* = 0.074) (Fig. S18).

In summary, our findings confirmed that BM-CAF-secreted IL26 and CX3CL1 promote cancer cell proliferation, invasion, migration, and therapeutic resistance through distinct mechanisms whereby IL26 activated the JAK1–STAT3 pathway, while CX3CL1 activated both JAK1–STAT3 and AKT–mTOR pathways, influencing EMT and CSC characteristics in NSCLC BM (Fig. 2O). A more detailed characterization of BM-CAF subpopulations using single-cell RNA-seq or lineage-tracing approaches would provide crucial insights into the cellular heterogeneity and functional diversity within the CAF compartment. Additionally, studies employing CRISPR-Cas9 genome editing could further validate these mechanistic findings with enhanced precision. Future validation of the clinical importance and immunologic aspect of these cytokines may establish targeting the IL26 and CX3CL1 signaling of BM-CAFs as a promising therapeutic strategy to overcome the current immunotherapy limitations in NSCLC BM.

## Supplementary Information

Below is the link to the electronic supplementary material.

Supplementary Material 1.

Supplementary Material 2.

Supplementary Material 3.

## Data Availability

The datasets used and analyzed during the current study are available from the corresponding author on reasonable request.

## Abbreviations

BP: Biological process
BM: Brain metastases
BM-CAF: Brain metastasis cancer-associated fibroblast
CAF: Cancer-associated fibroblast
CSC: Cancer stem cell
CC: Cellular component
CX3CL1: Chemokine (C-X3-C motif) ligand 1
DEG: Differentially expressed gene
EMT: Epithelial–mesenchymal transition
FACS: Fluorescence activated cell sorting
FAP: Fibroblast activation protein
IF: Immunofluorescences
IC_50_: Half maximal inhibitory concentration
IL26: Interleukin 26
JAK: Janus kinase
LLC1: Lewis lung carcinoma
MET: Mesenchymal–epithelial transition
MF: Molecular function
ML-CAF: Mouse lung cancer-associated fibroblast
ML-NF: Mouse lung normal fibroblast
NSCLC: Non-small cell lung cancer
NF: Normal fibroblast
PCA: Principal component analysis
RT: Radiation therapy
STAT: Signal transducers and activators of transcription
TME: Tumor microenvironment
