# Supplementary material for: Brain metastasis-associated cancer fibroblasts drive tumor progression and therapeutic resistance through IL26 and CX3CL1 signaling in non-small-cell lung cancer
Source: Exp Hematol Oncol. 2025 Sep 30;14:120. doi: 10.1186/s40164-025-00713-9 (PMC12482677; doi:10.1186/s40164-025-00713-9)
Supplement: Supplementary file 1 — Supplementary Material 1. [file 40164_2025_713_MOESM1_ESM.docx]

**Supplementary Material 1. to: Brain metastasis-associated cancer fibroblasts drive tumor progression and therapeutic resistance through IL26 and CX3CL1 signaling in non-small-cell lung cancer**

**Materials and Methods**

***Cell line and culture conditions***

Mouse Lewis lung carcinoma (LLC1), human NSCLC cell lines (A549 and H1975), human fibroblast (MRC5), and mouse fibroblast (NIH3T3) cell lines were obtained from American Type Culture Collection (ATCC). All cell lines were maintained in Dulbecco's Modified Eagle Medium (DMEM; Welgene, Gyeongsan, South Korea) supplemented with 10% fetal bovine serum (FBS) and 1% penicillin-streptomycin under standard culture conditions in a humidified incubator at 37°C with 5% CO₂.

***Patient-derived CAF and NF isolation and culture***

Fresh tumor specimens and scalp skin tissues were collected from NSCLC BM patients who underwent surgical resection at Chonnam National University Hwasun Hospital. Written informed consent was obtained from patients or their legal surrogates for the use of clinical information and tissue specimens. The study was approved by the Institutional Review Board of Chonnam National University Hwasun Hospital (CNUHH-2019–218). Patient-derived BM-CAFs and normal fibroblasts (NFs) were isolated from tumor and scalp skin tissues, respectively (Fig. 1A), following the previously established protocol [1]. Fresh tissue samples were minced into approximately one-millimeter fragments, and placed onto culture plates. After a brief adherence period of 5-10 min, the tissues were cultured in DMEM/Nutrient Mixture F-12 (DMEM/F-12) supplemented with 10% fetal bovine serum (FBS), 1% penicillin/streptomycin (P/S), and Normocin (100 μg/mL).

***Mouse fibroblast isolation and culture***

Mouse lung NFs (ML-NFs) were isolated from lung tissues of C57BL/6 mice (Supplementary Fig. 1A), following institutional animal care and use guidelines (CNU IACUC-H-2023-59). These fibroblasts were converted to cancer-associated fibroblasts (ML-CAFs) through co-culture with LLC1 cells using a transwell system (Supplementary Fig. 1B). All NFs and CAFs were maintained in a modified DMEM, termed F-Medium (Puricellmania, Seoul, South Korea) and used at or below passage 5.

***siRNA-mediated gene silencing***

To investigate the functional roles of IL26 and CX3CL1, small interfering RNAs (*si*RNAs) specifically targeting human IL26 (*si*IL26) and CX3CL1 (*si*CX3CL1) were purchased from Bioneer Corporation (Daejeon, South Korea). A non-targeting scrambled *si*RNA served as the negative control. Cells were seeded in 6-well plates at an appropriate density and transfected with *si*RNAs at a final concentration of 6 nM using Lipofectamine™ RNAiMAX Transfection Reagent (Thermo Fisher Scientific), following the manufacturer’s instructions. After 48–72 hours of transfection, cells were collected for downstream co-culture experiments with cancer cells. Knockdown efficiency of target genes was confirmed by Western blotting and RT-PCR prior to co-culture.

***Co-culture systems of NSCLC cells and CAFs/NFs***

The interactions between NSCLC cells and fibroblasts were studied using both indirect and direct co-culture methods. For indirect transwell co-culture experiments, A549 and H1975 NSCLC cells were seeded in the bottom chamber of six-well transwell plates (SPL life sciences, Pocheon, South Korea) at a density of 1 × 10^5^ cells per well. NFs and CAFs were seeded on transwell inserts with 0.4µm pore membranes, preventing direct contact between different cell types. The culture medium consisted of DMEM supplemented with 5% FBS, 1% penicillin-streptomycin, with 3 mL in the bottom chamber and 2 mL in the upper chamber. Co-cultures were maintained for 48 h under standard conditions.

For direct co-culture experiments, NSCLC cells were prelabeled with 5µM CellTracker Green 5-chloromethylfluorescein diacetate (CMFDA, Invitrogen life technologies, Carlsbad, CA, USA) in serum-free DMEM for 45 min at 37°C. The labeled NSCLC cells were then combined with NFs and CAFs and cultured in supplemented DMEM for 48 h under standard conditions.

***Cisplatin sensitivity assay***

NSCLC cells were seeded in 96-well plates at densities ranging from 5,000 to10,000 cells per well and incubated for 24 h. Cells were then exposed to increasing concentrations of cisplatin (0 µg/mL, 1.25 µg/mL, 2.5 µg/mL, 5 µg/mL, 10 µg/mL, 20 µg/mL, and 40 µg/mL) for 48 h to determine the IC_50_ value. To investigate the role of IL26 and CXCL1 in BM-CAF conditioned medium on cisplatin resistance, cells were treated with either neutralizing antibodies or *si*RNA targeting IL26 and CX3CL1. Cell viability was evaluated using the Post-treatment, 10 µL of EZ-Cytox solution (EZ-Cytox Cell Viability Assay Kit (Cat No. EZ-1000, DoGenBio, Seoul, Korea) according to manufacturer instructions. Following the addition of EZ-Cytox solution 10 µL, plates were incubated for 1-3 h, and absorbance was measured at 450 nm using a microplate reader. All experimental conditions were performed in triplicate.

***Cell proliferation assay***

NSCLC cells (A549 and H1975) were seeded at a density of 3 × 10⁴ cells per well in the bottom chamber of 24-well transwell plates (SPL Life Sciences), while NFs and BM-CAFs were seeded at a density of 6 × 10⁴ cells per insert. Fibroblastss were stabilized for 48 h, and NSCLC cells were incubated for 24 h under standard conditions. After phosphate-buffered saline washing, NSCLC cells were serum-starved to synchronize cell cycles. The bottom and upper chambers were then filled with supplemented DMEM and incubated for up to 48 h. Cell proliferation was assessed by Ki-67 immunofluorescence staining using specific antibodies (clone SP6, ab16667, Abcam, Waltham, MA, USA).

***Transwell invasion assay***

Invasion assay was performed using a 24-well transwell plate (Corning Inc. Corning, NY, USA) with gelatin-coated inserts. Fibroblasts were seeded in the bottom chamber at a density of 5 × 10^4^ cells/well in supplemented DMEM. After twenty-four hours, NSCLC cells were seeded onto the insert (8 µm pore size) at a density of 1 × 10^4^ cells per well in serum-free DMEM for 2-3 h. Following cell adhesion, the inserts containing the NSCLC cells were transferred to the lower chambers containing fibroblasts. The co-culture system was maintained in low-serum conditions for up to 48 hours. Invasive cells were fixed with methanol for 5 min, stained using the Diff-Quik staining method, and quantified using ImageJ software.

***Migration assay***

NSCLC cells and fibroblasts were seeded at a density of 1 × 10^5^ cells/well in their respective chambers and cultured independently for 48 h. Upon reaching 100% confluency, NSCLC cells were serum-starved for twelve hours, and a linear scratch was created using a pipette tip. Fibroblast-containing inserts were positioned above the scratched cancer cells, and the co-culture was maintained in supplemented DMEM. Wound closure were monitored at 0, 24, and 48 h using phase-contrast microscopy, with migration quantified by measuring the scratch width reduction.

***Flow cytometry analysis***

Chemoresistance of NSCLC cells to cisplatin following BM-CAF exposure was evaluated using flow cytometry at cisplatin concentration of 2.5 µg/ml and 5 µg/ml, respectively. Neutralizing antibody or *si*RNA against IL26 and CX3CL1 was treated under direct co-culture system in BM-CAF induced cisplatin sensitivity. Apoptotic responses were quantified using the APC Annexin V Apoptosis Detection Kit with Propidium Iodide (PI) (BioLegend, San Diego, CA, USA), according to the manufacturer’s protocol. Cell populations were analyzed using a BD FACSCanto™ II flow cytometer (BD Biosciences, San Jose, CA, USA), with APC-conjugated Annexin V and Propidium Iodide detected via APC and PE channels, respectively. CellTracker Green-labeled cells stained with 5-Chloromethylfluorescein diacetate (CMFDA) were detected through the FITC channel. Data acquisition and analysis were performed according to standardized flow cytometry protocols FlowJo™ v10.8 Software (BD Life Sciences) to quantify apoptotic cell population.

***Immunofluorescence (IF) staining***

Fibroblasts were cultured in 8-well Lab-Tek II Chamber Slide system (Cat No. 154534, Thermo Scientific, Waltham, MA, USA) at a density of 5 × 10^3^ cells/well overnight under standard conditions. Cells were fixed with 4% paraformaldehyde, permeabilized with 0.1% Triton X-100 (Sigma-Aldrich Life Science, Munich, Germany‎), and blocked with 2% bovine serum albumin (BSA) (BioShop Canada Inc., Burlington, ON, Canada) in Tris-buffered saline with Tween 20 (TBST). Primary antibodies (Supple Table 4) targeting α-SMA, PDGFR-β, and pan-Keratin were applied overnight, followed by Alexa 488 (1:500, Cat No. A11001, Thermo Scientific) and Alexa 568 (1:500, Cat No. A11077, Thermo Scientific) secondary antibodies. Cell nuclei were counterstained with 4', 6-diamidino-2-phenylindole (DAPI) (1:1000, 0.1µg/mL). Mounted specimens were visualized using confocal microscopy (EVOS FL system, Thermo Scientific) at appropriate magnifications.

Tumor tissues from mice injected with A549 cells (and/or NF or BM-CAFs) were fixed in 10% formalin and embedded in paraffin. Tissue sections were cut at a thickness of 4 µm and mounted onto slides. Deparaffinization was performed using xylene followed by a graded series of ethanol. Antigen retrieval was carried out in 10 mmol/L citrate buffer (pH 6.0) by heating the sections at 95 °C for 15 minutes. The tissue sections were then permeabilized with 0.1% Triton X-100 for 10 minutes and endogenous peroxidase activity was quenched using hydrogen peroxide (H₂O₂, DAKO S2023) for 20 minutes at room temperature. Slides were incubated overnight at 4 °C with the following primary antibodies: SLUG (1:200, Cat. No. 9585, Cell Signaling Technology), Vimentin (1:250, Cat. No. ab8978, Abcam), and CD44 (1:200, Cat. No. ab189524, Abcam). The next day, tissue sections were incubated with Alexa Fluor 488 or Alexa Fluor 568-conjugated secondary antibodies. Cell nuclei were counterstained with DAPI and mounted specimens were visualized using confocal microscopy.

***Western blot analysis***

Western blot analysis was performed to evaluate protein expression as described previously [2]. Primary and secondary antibodies used in the current study are listed in Supplementary Table 4.

***Reverse transcription(RT)-PCR and Quantitative RT-PCR (qRT PCR)***

Gene expression was assessed using RT-PCR and qRT-PCR as described previously [2]. Primer sequences used in the current study are listed in Supplementary Table 5.

***Cytokine array assay***

Cytokine secretion profiles in BM-CAFs and ML-CAFs were analyzed using the Proteome Profiler Human and Mouse XL Cytokine Array Kits (R&D Systems Inc., Minneapolis, MN, USA) following manufacturer's instructions.

***RNA sequencing, differentially expressed gene (DEG) and pathway enrichment analysis***

RNA sequencing (RNAseq) was performed on paired samples of patient-derived BM-CAFs and NFs. RNA extraction and sequencing data generation were outsourced to Theragen Etex (Seoul, South Korea). Data analysis was conducted using R software (R Development Core Team, Vienna, Austria) with the DESeq2 package for normalization and differential expression analysis. Differentially expressed genes (DEGs) were identified based on adjusted p-value (< 0.05) and absolute log2 fold change (> 1). Gene symbols were converted to Entrez IDs using the *bitr* function from the *clusterProfiler* package. Visualization of differential expression was performed using the Enhanced Volcano package, while other graphical presentations were generated using the *ggplot2* package. For correlation analysis, a matrix was computed to assess relationships between gene expression profiles across samples. Pearson correlation coefficients were calculated using the *cor* function in R. A heatmap of the correlation matrix was generated using the *pheatmap* package, with hierarchical clustering applied to both rows and columns based on Euclidean distance. Sample metadata was incorporated as annotations with predefined color coding for different groups. Principal component analysis (PCA) was performed to visualize variance in gene expression data among samples. The transformed DESeq2 dataset was transposed and scaled before applying PCA using the *prcomp* function in R. The first two principal components (PC1 and PC2) were extracted and visualized using the *ggbiplot* package. Sample groupings were overlaid using metadata from a corresponding sample information file. Ellipses were drawn to indicate group clustering.

Gene Ontology (GO) enrichment analysis was conducted using the enrichGO function, with annotations from the org.Hs.eg.db database. The analysis covered Biological Process (BP), Cellular Component (CC), and Molecular Function (MF) ontologies, with p-value and q-value cutoffs of 0.05. KEGG pathway enrichment analysis was conducted using the *enrichKEGG* function with p-value cutoffs of 0.1. Both GO and KEGG results were visualized using dot plots, where enriched terms were ranked based on adjusted p-values, and gene counts were represented by point sizes. Clustering analysis was performed using the online bioinformatics tool iDEP software ver. 96 [3], followed by KEGG pathway enrichment analysis focusing on cytokine-cytokine receptor interaction. Selected cytokine-cytokine receptor interaction gene expression levels (log2FoldChange) were visualized in a heatmap by using *pheatmap* function in R.

***Enzyme-linked immunosorbent assay (ELISA)***

Secreted levels of IL26 and CX3CL1 in cell culture supernatants from patient-derived BM-CAFs were quantified using Quantikine ELISA Kits (R&D Systems, Minneapolis, MN, USA) according to manufacturer protocols.

***Cytokine neutralizing study***

The functional significance of BM-CAF-secreted cytokines was evaluated using neutralizing antibodies against human IL26 (anti-*h*IL26) and human CX3CL1 (anti-*h*CX3CL1). Anti-*h*IL26 antibody (2 µg/mL; Cat No. AF1375, R&D Systems, USA) and anti-*h*CX3CL1 antibody (1 µg/mL; Cat No. AF365, R&D Systems, USA) were added to the co-culture system to inhibit cytokine activity. The effects of cytokine neutralization on lung cancer cell behavior were assessed using established co-culture methods.

***Immunohistochemical staining***

Formalin-fixed, paraffin-embedded NSCLC BM tissues were reviewed on hematoxylin and eosin (H&E)-stained sections to identify representative blocks for further analysis. From these paraffin blocks, 3-μm thick sections were prepared and subjected to immunohistochemical (IHC) staining using a Bond-Max automated stainer (Leica Microsystems, Buffalo Grove, IL, USA) following standard protocols, as previously described [1]. The primary antibodies used were anti-CX3CL1 (1:500 dilution; catalog no. HPA040361; Sigma-Aldrich, St. Louis, MO, USA) and anti-IL26 (1:3000 dilution; catalog no. ab224198; Abcam, Cambridge, UK). All stained slides were independently reviewed by two experienced pathologists (SSK and KHL) blinded to the patients' clinical data. Expression levels of IL26 and CX3CL1 in BM-CAFs were semi-quantitatively categorized into two groups: low expression and high expression.

***Animal model***

Male C57BL/6 and BALB/c-nu nude mice (G-bio Company, Busan, Korea) aged 6-8 weeks were used for the studies. The experimental protocol was approved by the Chonnam National University Medical School Research Institutional Animal Care and Use Committee (CNU IACUC-H-2023-59), and all animal experiments were conducted following recognized principles for animal management and use (DHU publication, NIH 80-23).

For subcutaneous tumor models, 1 × 10⁵ LLC1 cells were subcutaneously injected into C57BL/6 group (*N=4*), either alone or with ML-NF or ML-CAF at a 1:1.5 ratio. Treatment with cisplatin (5 mg/kg body weight) was commenced when tumor volumes exceeded 450 mm³. In the xenograft model, 1 × 10⁶ A549 cells were injected into BALB/c-nu nude mice (*N=4*), either alone or with patient-derived fibroblasts at a 1:0.5 ratio (5 × 10⁵ cells/mouse). Cisplatin treatment (5 mg/kg body weight) began when tumor volumes reached 50 mm³. Tumor volumes were measured daily using calipers and calculated using standard formulae: Tumor volume = (Length × Width²) / 2. Tissues were collected for analysis when tumors reached 1500 mm³.

An intracranial xenograft model was established using stereotactic injection techniques, as previously describe method [2]. A 1 mm burr hole was created at 2 mm lateral to the sagittal suture and 2 mm posterior to the coronal suture in the right hemisphere of BALB/c-nu nude mice. A549 cells (1 × 10^6^) were injected into the brain using a 26-gauge Hamilton syringe at a depth of 3 mm, either alone or with fibroblasts at a 1:0.5 ratio, under the guidance of a stereotactic apparatus (KDS310, KD Scientific, Holliston, MA, USA). Tumor progression was monitored using magnetic resonance imaging with a 3 Tesla MRI scanner (Skyra Atim System, Siemens, Erlangen, Germany) equipped with a 3 cm diameter radiofrequency coil (Stark Contrast, Erlangen, Germany). Multi-slice coronal images were acquired using a fast spin echo sequence, as described previously [2].

To validate the tumor-promoting functions of IL-26 and CX3CL1, an additional xenograft mouse model was established. A549 cells were co-injected subcutaneously with BM-CAFs at a 1:0.5 ratio. Three experimental groups were established, each consisting of six mice. The control group (A549:BM-CAF) received intraperitoneal injections of isotype IgG (2–4 µg in 100 µL PBS per mouse). The two treatment groups received either anti-IL-26 antibody (2 µg in 100 µL PBS per mouse) or anti-CX3CL1 antibody (4 µg in 100 µL PBS per mouse) to evaluate the neutralizing effects of IL-26 and CX3CL1 secreted from CAFs on tumor progression. Antibody treatments were initiated on the second day after cell injection and administered every other day for a total of six doses. Tumor growth was monitored to assess the efficacy of cytokine neutralization in reducing tumor burden.

***Statistical Analysis***

Experimental data were analyzed using GraphPad Prism software (version 8.01, GraphPad Software Inc., San Diego, CA, USA). Results are presented as the mean ± standard error of the mean (SEM). Statistical comparisons between groups were performed using appropriate parametric tests, including Student’s t-test and analysis of variance (ANOVA) with post-hoc corrections for multiple comparisons.

The criteria for recurrence-free survival (RFS) and overall survival (OS) periods were defined according to our previously published methodology [4]. RFS and OS were investigated in an in-house cohort of 68 patients with NSCLC brain metastasis. The study was approved by the Institutional Review Board of Chonnam National University Hwasun Hospital (CNUHH-2024-114). The effects of IL26 and CX3CL1 expression levels on RFS and OS were determined using the Kaplan–Meier method with log-rank tests. Survival comparisons were analyzed using SPSS version 30.0 software for Windows (SPSS Inc., Chicago, IL, USA). Statistical significance was established at *P* < 0.05.

**References:**

1. Akanda MR, Ahn EJ, Kim YJ, Salam SMA, Noh MG, Kim SS, et al. Different Expression and Clinical Implications of Cancer-Associated Fibroblast (CAF) Markers in Brain Metastases. Journal of Cancer. 2023;14(3):464-79.

2. Ahn EJ, Kim YJ, Akanda MR, Oh SJ, Jung TY, Jung S, et al. Metastasis-enhancing protein KITENIN confers temozolomide resistance on glioblastoma with unmethylated MGMT via upregulation of cancer stem cell makers. Clin Transl Med. 2024;14(8):e1804.

3. Ge SX, Son EW, Yao R. iDEP: an integrated web application for differential expression and pathway analysis of RNA-Seq data. BMC Bioinformatics. 2018;19(1):534.

4. Akanda MR, Ahn EJ, Kim YJ, Salam SMA, Noh MG, Lee TK, et al. Analysis of stromal PDGFR-β and α-SMA expression and their clinical relevance in brain metastases of breast cancer patients. BMC Cancer. 2023;23(1):468.
