# Supplementary material for: Brain metastasis-associated cancer fibroblasts drive tumor progression and therapeutic resistance through IL26 and CX3CL1 signaling in non-small-cell lung cancer
Source: Exp Hematol Oncol. 2025 Sep 30;14:120. doi: 10.1186/s40164-025-00713-9 (PMC12482677; doi:10.1186/s40164-025-00713-9)
Supplement: Supplementary file 2 — Supplementary Material 2. [file 40164_2025_713_MOESM2_ESM.docx]

**Supplementary Material 2. to: Brain metastasis-associated cancer fibroblasts drive tumor progression and therapeutic resistance through IL26 and CX3CL1 signaling in non-small-cell lung cancer**

**Supplementary Figures and Legends**


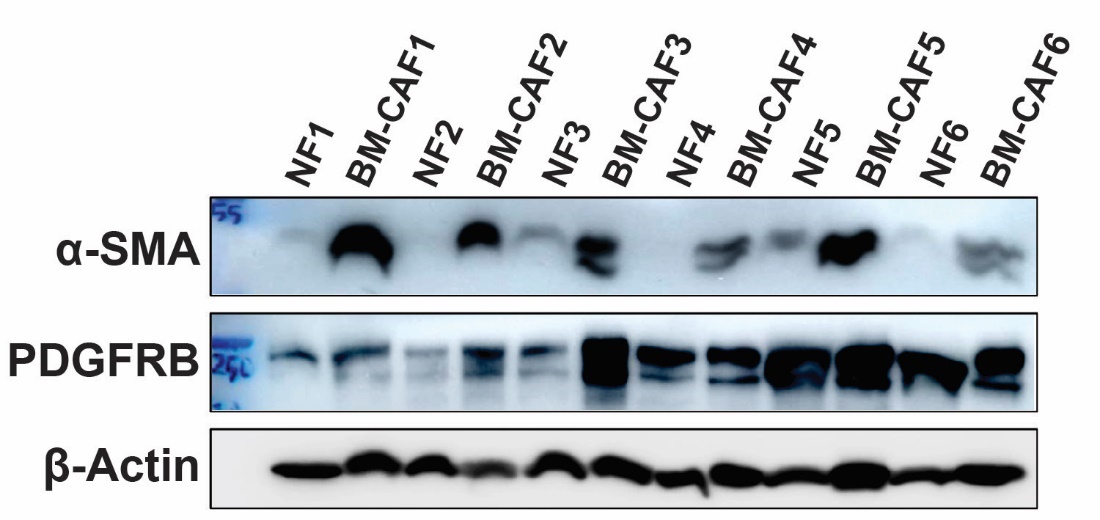


**Supplementary Figure 1. Validation of α-SMA and PDGFR-β protein expressions in patient-derived BM-CAF.**

Western blot analysis of paired NFs and BM-CAFs (n=6) derived from NSCLC BM patients demonstrates that BM-CAFs consistently exhibited elevated expression of CAF markers α-SMA and PDGFR-β compared to matched NFs.


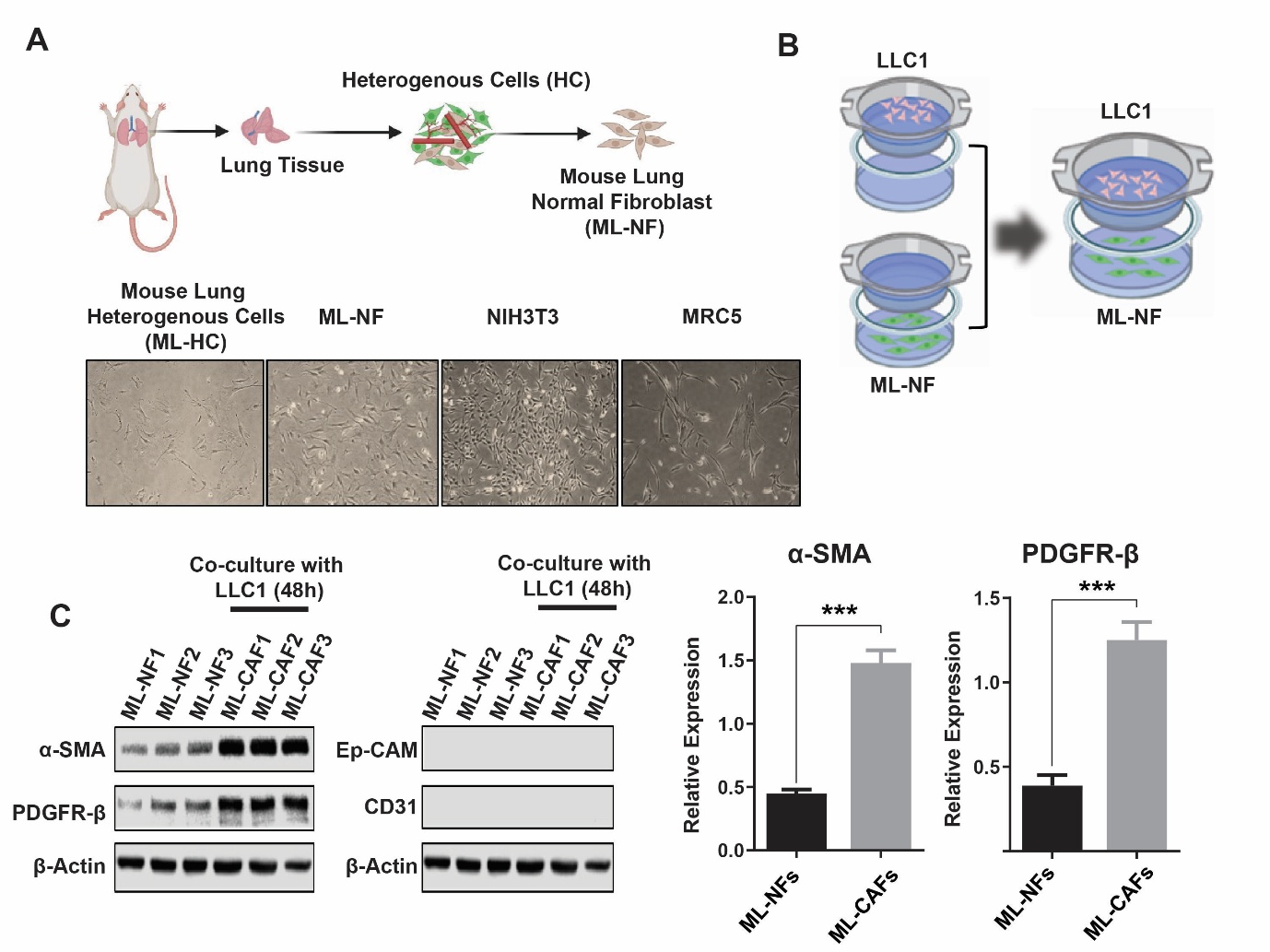


**Supplementary Figure 2. Isolation and characterization of ML-NFs and induction of ML-CAFs through co-culture system.**

(A) Phase-contrast microscopy of ML-NFs isolated from lung tissue demonstrated a uniformly spindle-shaped morphology comparable to NIH3T3 and MRC5 fibroblast cell lines. (B) A transwell co-culture system was established using LLC1 and ML-NFs for all subsequent experiments. (C) Western blot analysis following co-culture revealed significant upregulation of α-SMA and PDGFR-β expression in ML-CAFs compared to ML-NFs, while negative markers Ep-CAM and CD31 remained undetectable in both fibroblast populations. Protein expression levels were normalized to β-Actin. Data are presented as means ± SEM with statistical significance indicated at ****P*<0.001.


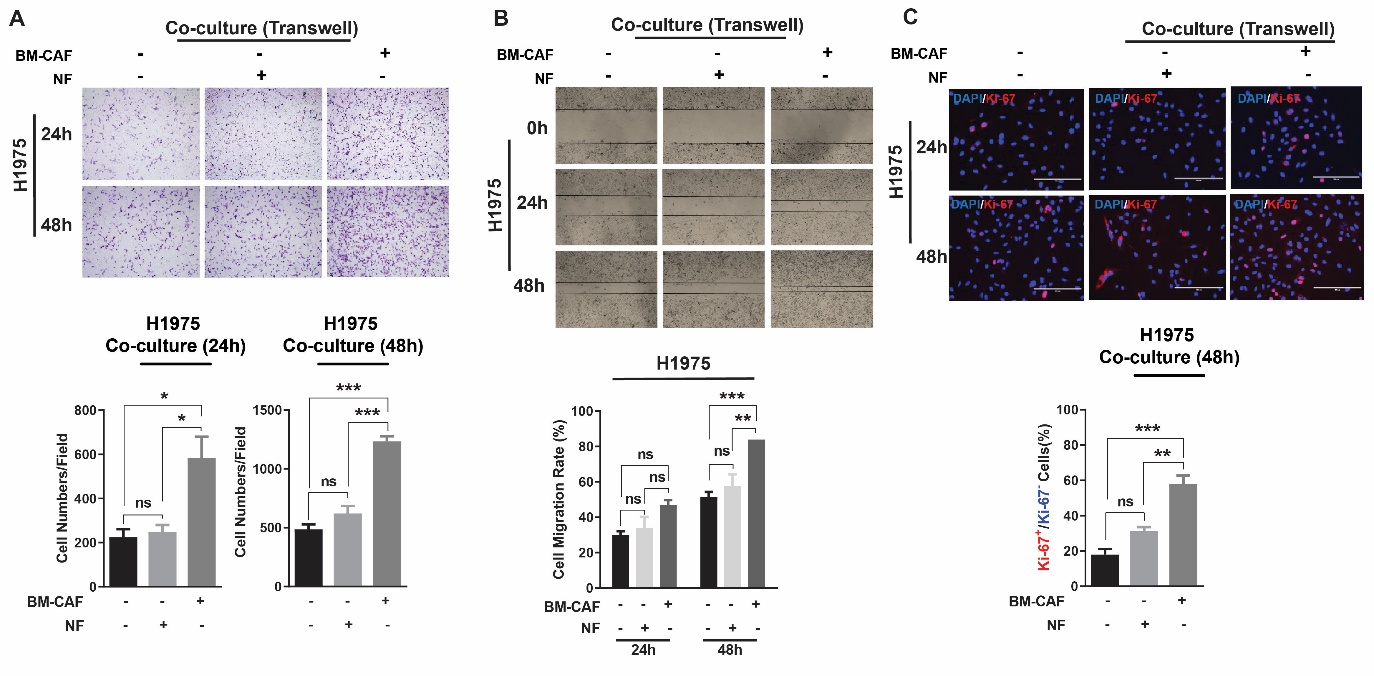


**Supplementary Figure 3.** **BM-CAFs significantly enhanced lung cancer cells invasion, migration and proliferation.**

(A) BM-CAFs enhanced the invasive capacity of NSCLC cell lines H1975 at both 24 h and 48h. (B) BM-CAFs promoted migration of H1975 cells at 48 h.(C) Cell proliferation analysis using Ki-67 labeling (*red*) demonstrated significantly increased proliferation of H1975 cells when co-cultured with BM-CAFs for 48 h compared to control or NFs co-culture conditions. The results are represented as mean ± SEM, based on one-way and two-way ANOVA analysis, followed by post hoc correction with Tukey’s and Bonferroni’s multiple comparison with statistical significance indicated at **P*<0.05, ***P*<0.01, and ****P*<0.001.

**
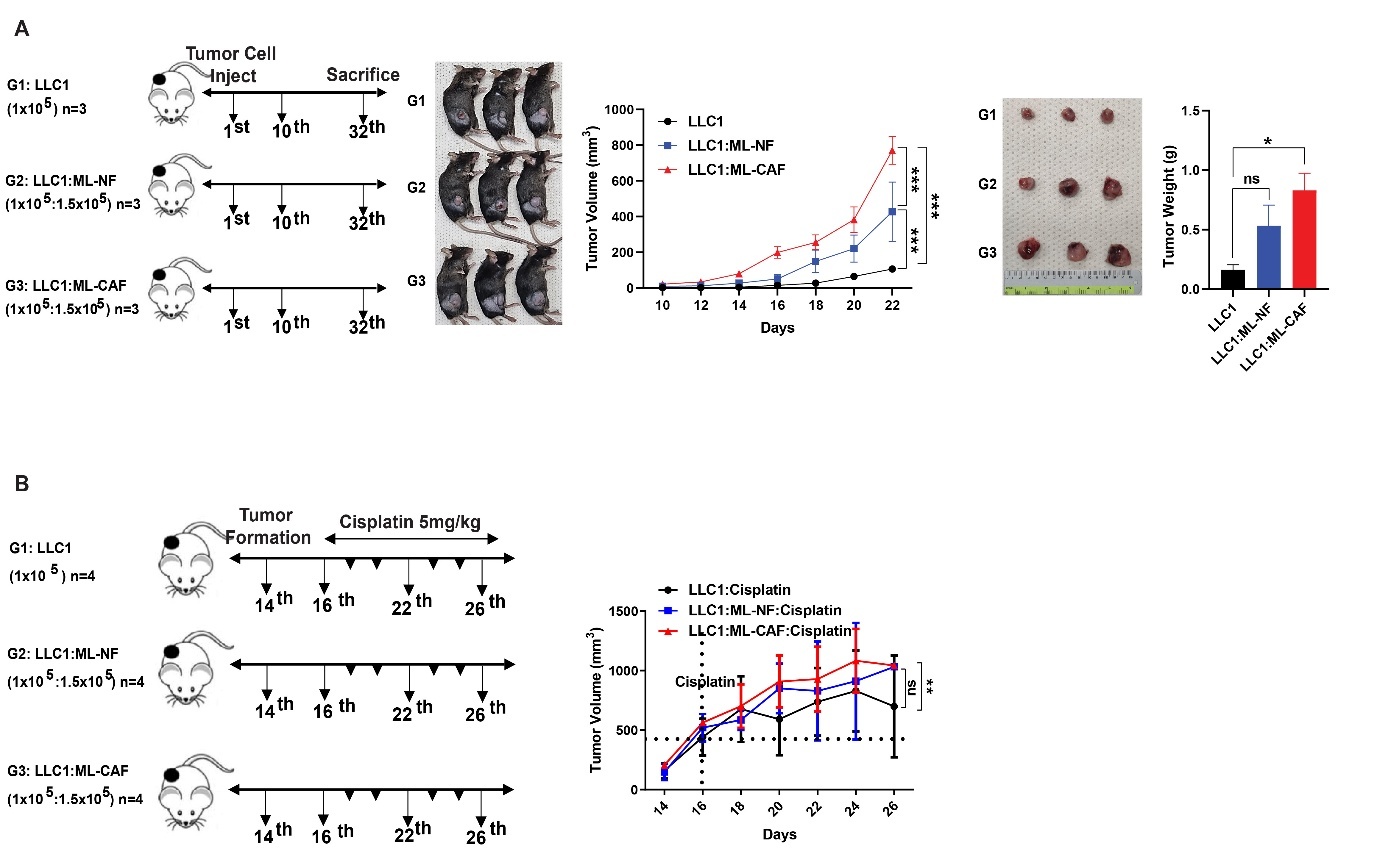
**

**Supplementary Figure 4. ML-CAFs enhance tumor growth and confer cisplatin resistance in a mouse xenograft model.**

(A) Experimental groups (*N*=3 per group) consisted of LLC1 cells alone or co-injected with either ML-NFs or ML-CAFs at a 1:0.5 ratio. Representative tumor images and temporal progression of tumor volume demonstrated significantly larger tumors in the ML-CAFs co-injection group, with higher tumor weight observed at experimental endpoint. (B) Following cisplatin treatment initiation on day 16, tumor volume progression showed sustained increase of tumor growth in the ML-CAF group compared to the LLC-alone group, despite cisplatin treatment, with tumors exceeding the 450 mm³ threshold (indicated by dotted line). Data are presented as means ± SEM with statistical significance indicated at **P*<0.05, ***P*<0.01, and ****P*<0.001.


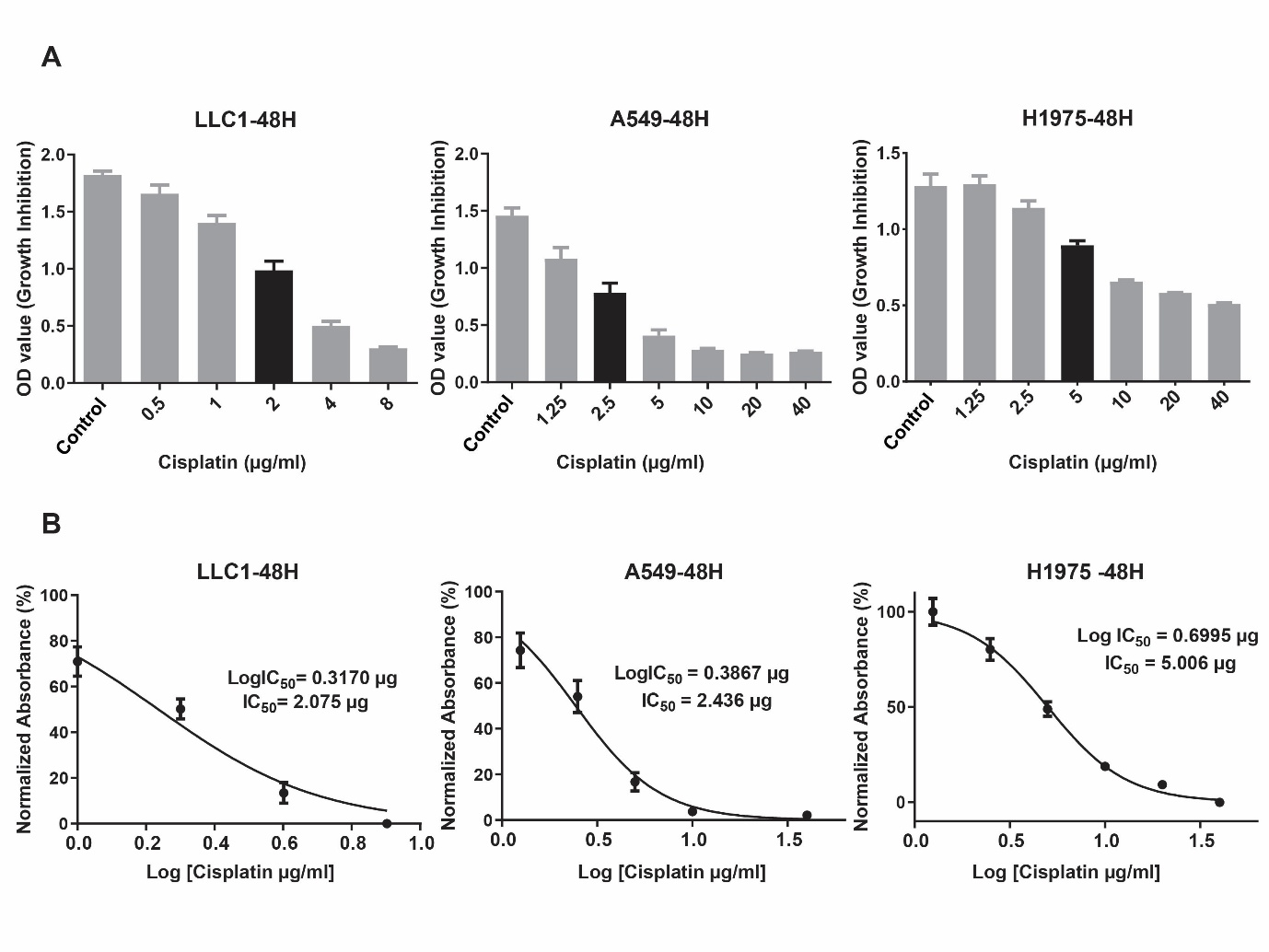


**Supplementary Figure 5. Dose-dependent growth inhibition and IC_50_ determination of cisplatin in LLC1, A549, and H1975 cells.**

(A) Growth inhibition responses to cisplatin were evaluated in LLC1, A549, and H1975 cells following 48-h treatment with increasing cisplatin concentrations. Cell growth inhibition was assessed by optical density measurements across different cisplatin doses, and corresponding dose-response curves were generated. (B) Logarithmic transformation of cisplatin concentrations revealed IC_50_ values of 2.075 µg/ml (~2.0 µg/ml) for LLC1, 2.436 µg/ml (~2.5 µg/ml) for A549, and 5.006 µg/ml (~5.0 µg/ml) for H1975 cells, demonstrating differential sensitivity to cisplatin treatment among the cell lines.


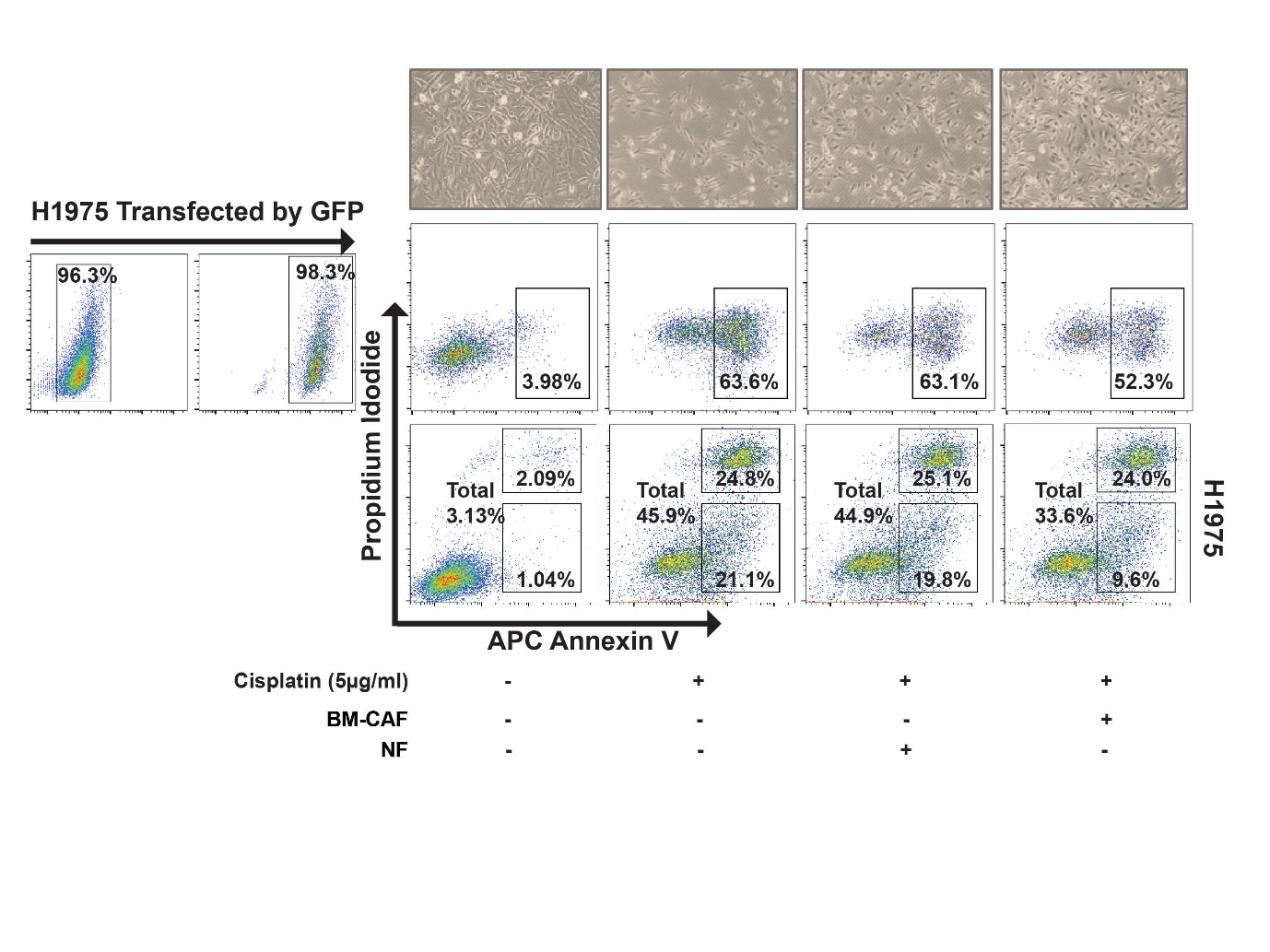


**Supplementary Figure 6. BM-CAFs attenuate cisplatin-induced apoptosis in H1975 cells.**

FACS analysis revealed that co-culture with BM-CAFs significantly reduced the proportion of cisplatin-induced apoptotic H1975 cells compared to both cisplatin-alone and NFs co-culture conditions. This protective effect was consistently observed in both direct and indirect transwell co-culture experimental settings.


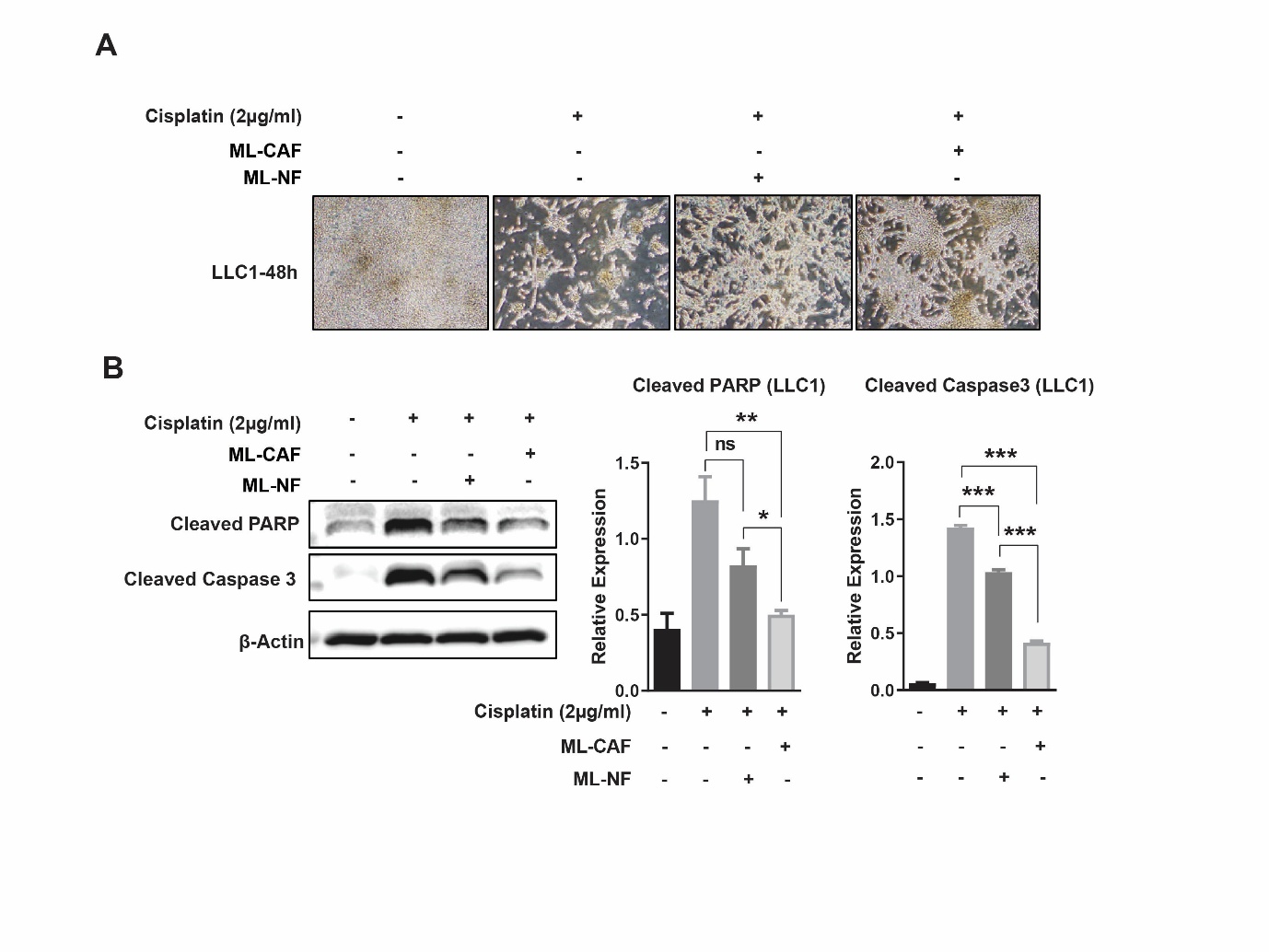


**Supplementary Figure 7. ML-CAFs reduce cisplatin-induced apoptosis in LLC1 cells.**

(A) Representative images demonstrated reduced cisplatin sensitivity in LLC1 cells when co-cultured with ML-CAFs compared to LLC1-alone or ML-NFs co-culture conditions. (B) Western blot analysis revealed significantly decreased expression of apoptotic markers cleaved PARP and cleaved caspase-3 in LLC1 cells co-cultured with ML-CAFs compared to both LLC1-alone or ML-NFs co-culture groups, indicating that ML-CAFs-mediated protection against cisplatin-induced apoptosis. Data are presented as means ± SEM with statistical significance indicated at **P*<0.05, ***P*<0.01, and ****P*<0.001.


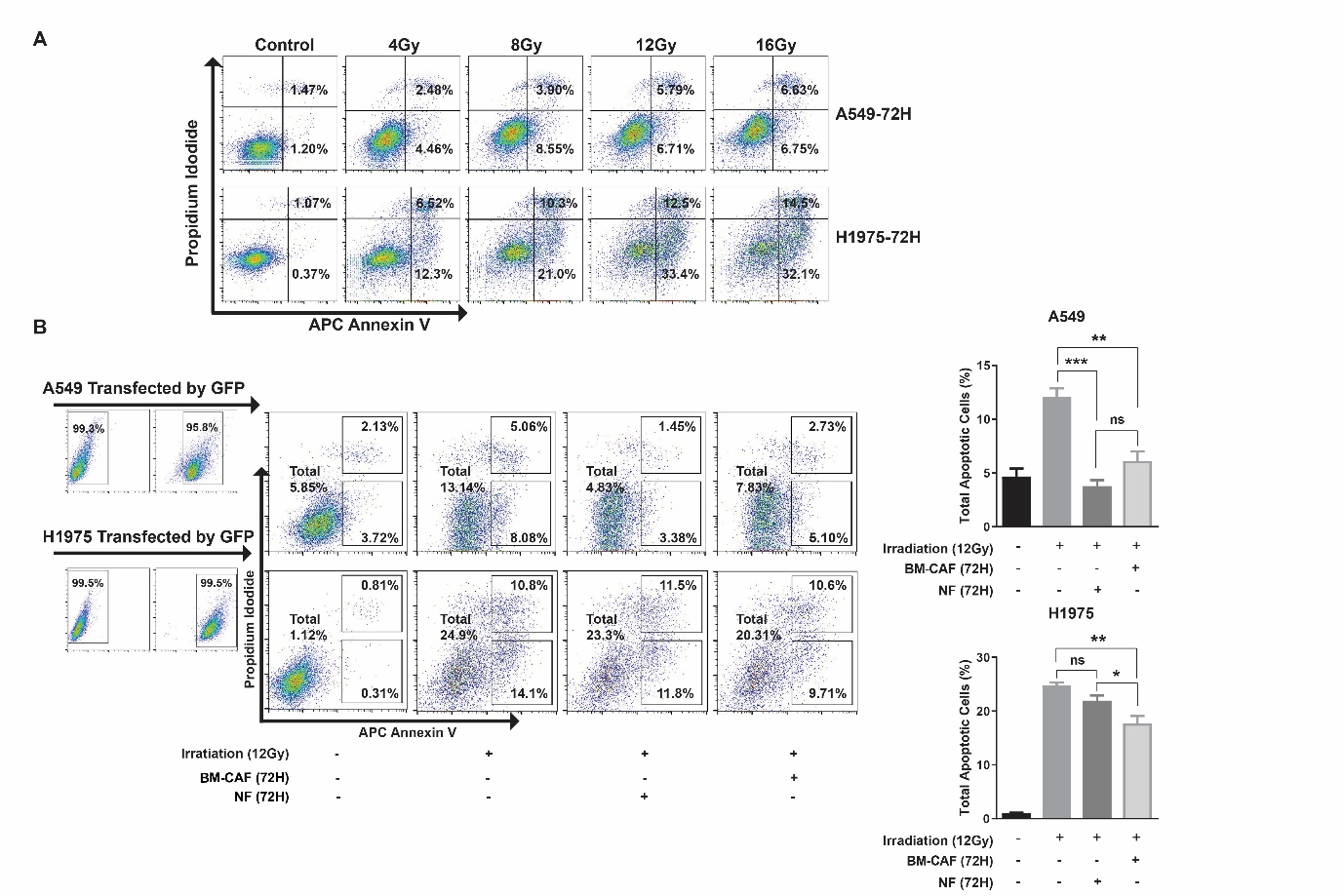


**Supplementary Figure 8. BM-CAFs attenuate irradiation-induced apoptosis in NSCLC cells.**

(A) FACS analysis was performed to evaluate irradiation-induced cell death at 72 h post-treatment across various radiation doses, with 12 Gy selected for the experimental conditions. (B) Further FACS analysis revealed that co-culture with BM-CAFs resulted in a modest reduction in the proportion of apoptotic cells compared to control conditions. Data are presented as means ± SEM with statistical significance indicated at **P*<0.05, ***P*<0.01, and ****P*<0.001.


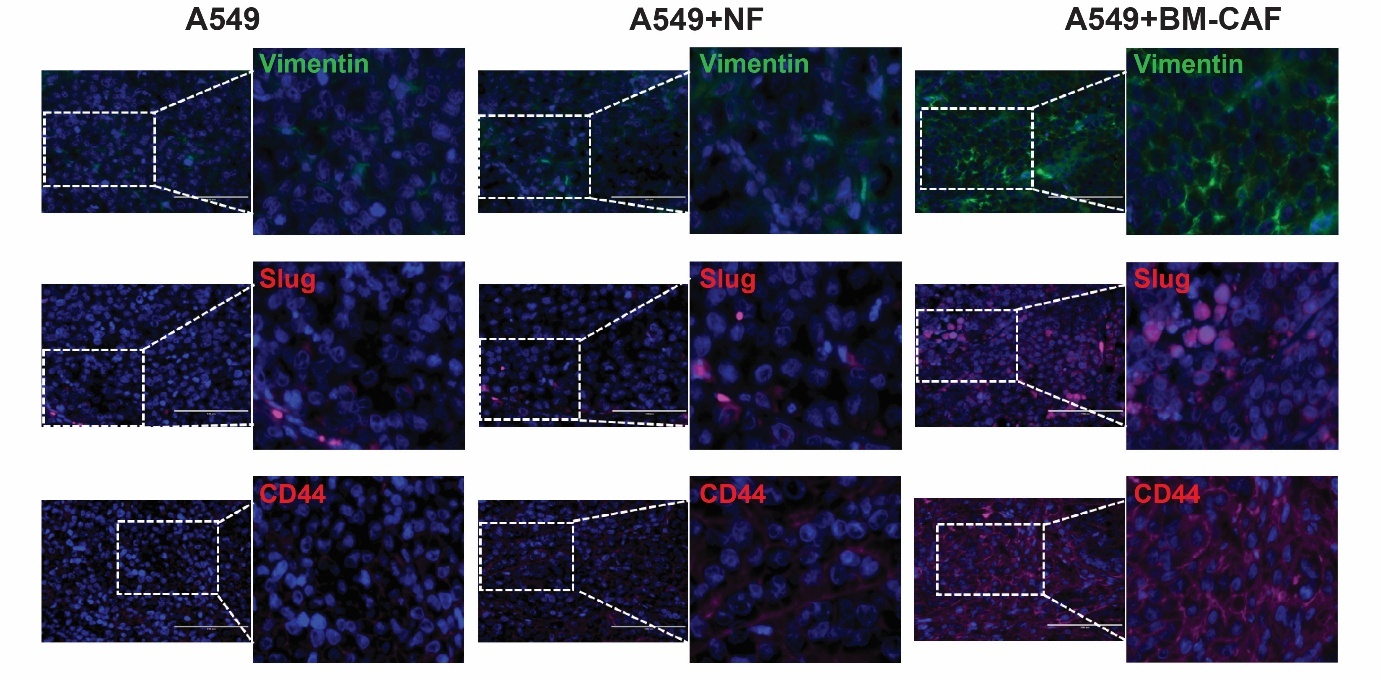


**Supplementary Figure 9. BM-CAFs enhance EMT and CSC marker expression in tumor tissues from the A549 xenograft mouse model.**

Immunofluorescence staining of Slug (red), Vimentin (green), and CD44 (red) in formalin-fixed, paraffin-embedded tumor sections from A549 only, A549+NF, and A549+BM-CAF co-injected xenografts. Tumors from the A549+BM-CAF group exhibited markedly elevated expression of Slug, Vimentin, and CD44 compared to A549 alone or A549+NF groups, indicating enhanced EMT and CSC traits. Dashed white boxes indicate magnified regions.


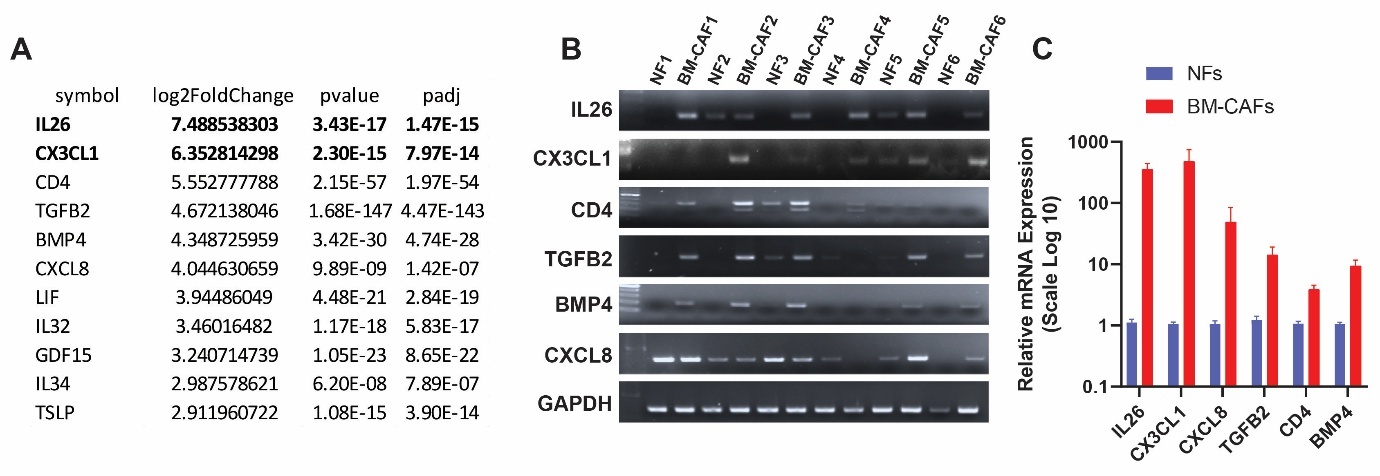


**Supplementary Figure 10. BM-CAF transcriptomic profiling identifies IL26 and CX3CL1 as top upregulated cytokines in BM-CAFs, validated across patient-derived samples.**

(A) Differential expression analysis of cytokine genes in BM-CAFs compared to NFs from RNA sequencing samples. The table displays the top upregulated cytokines with corresponding log₂ fold change, p-values, and adjusted p-values (padj). IL26 and CX3CL1 showed the highest log₂ fold changes with strong statistical significance. (B) RT-PCR validation of IL26, CX3CL1, and other cytokines across six paired NF and BM-CAF samples from isolated cultured cells. GAPDH was used as a loading control. (C) Quantitative RT-PCR confirming elevated mRNA expression levels of IL26 and CX3CL1 in BM-CAFs compared to NFs from isolated cultured samples. Data are presented as mean ± SEM from three independent experiments.


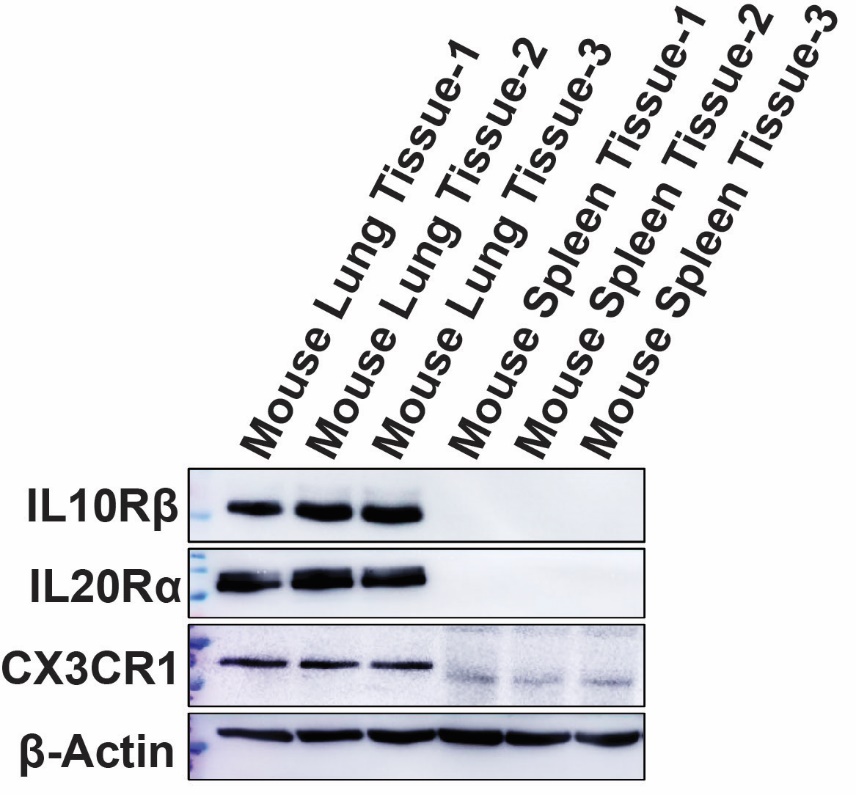


**Supplementary Figure 11. Western blot analysis of IL26 and CX3CL1 receptor expression in tissues used as positive and negative controls.**

IL10Rβ, IL20Rα, and CX3CR1 expression was assessed in mouse lung tissue (positive controls) and mouse spleen tissue (negative controls).


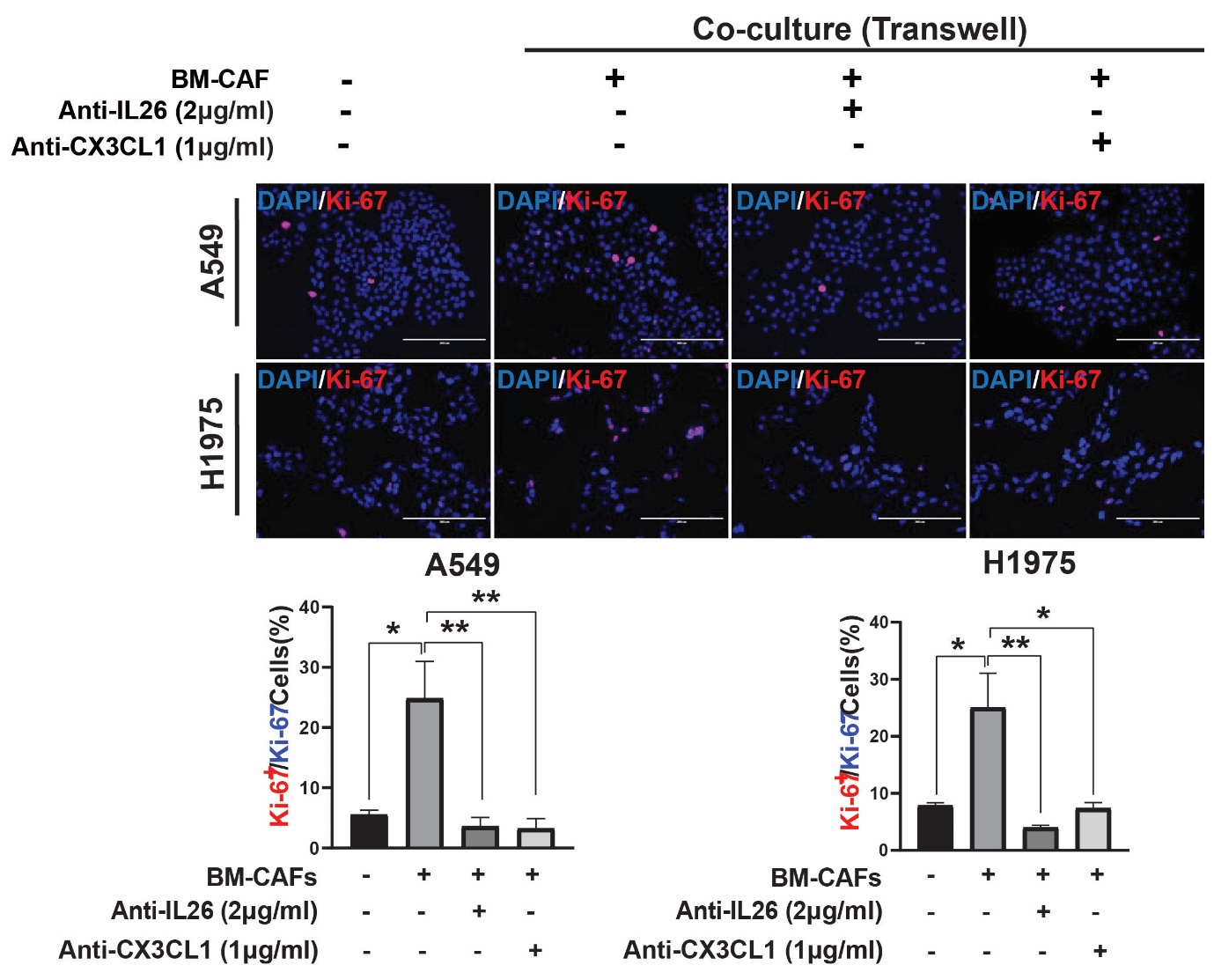


**Supplementary Figure 12.** **Neutralization of IL26 and CX3CL1 suppresses BM-CAF induced proliferation of lung cancer cells in a transwell co-culture system.**

Immunofluorescence staining for Ki-67 (red) in A549 and H1975 cells co-cultured with BM-CAFs in a transwell system, with or without anti-IL26 or anti-CX3CL1 neutralizing antibodies. Quantification (bottom panels) shows significant reduction in Ki-67⁺ proliferating cells upon neutralization of IL26 or CX3CL1. Data are shown as mean ± SEM from three independent experiments (*P* < 0.05, *P* < 0.01; one-way ANOVA with Tukey’s post hoc test).


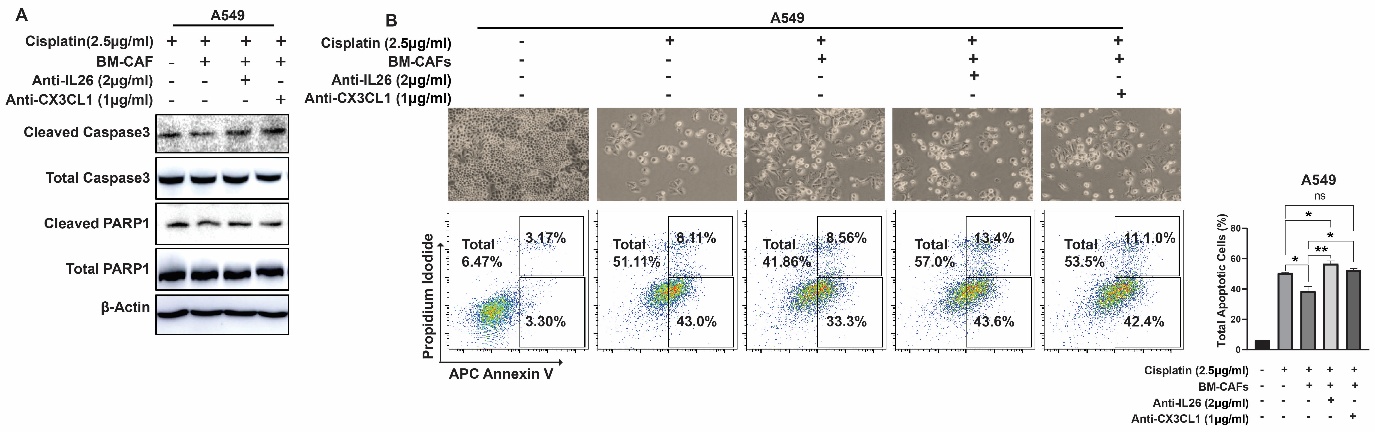


**Supplementary Figure 13. Neutralization of IL26 and CX3CL1 in BM-CAFs enhances cisplatin sensitivity in lung cancer cells.**

(A) Western blot analysis of A549 cells co-cultured with BM-CAFs in the presence of cisplatin and treated with neutralizing antibodies against IL26 or CX3CL1. Neutralization of IL26 and CX3CL1 restored cisplatin sensitivity, as evidenced by increased expression of cleaved caspase-3 and cleaved PARP1, indicating enhanced apoptosis. (B) Flow cytometry analysis showing increased total apoptotic cell populations following anti-IL26 (57%) and anti-CX3CL1 (53.5%) treatment in the BM-CAF cisplatin co-culture group, compared to BM-CAF cisplatin (41.86%) groups. Notably, anti-IL26 treatment led to a significant increase in apoptosis compared to cisplatin alone. All data represent mean ± SEM from three independent experiments. *P* < 0.05, *P* < 0.01.


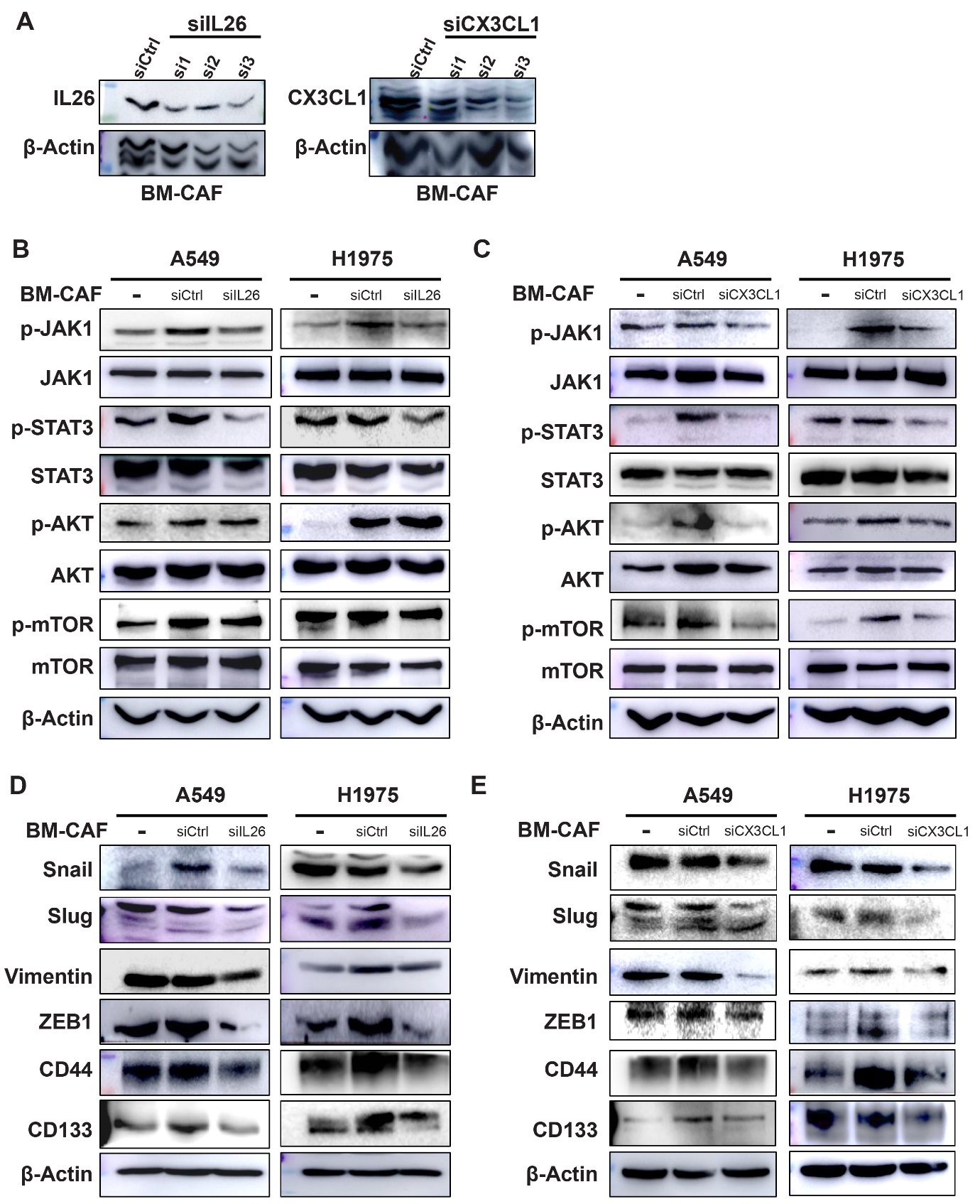


**Supplementary Figure 14. siRNA-mediated knockdown of IL26 and CX3CL1 in BM-CAFs suppresses oncogenic signaling and EMT/CSC marker expression in lung cancer cells.**

(A) Western blot analysis confirming knockdown efficiency of IL26 (left) and CX3CL1 (right) in BM-CAFs transfected with three independent siRNAs (si1, si2, si3) targeting each gene, compared to non-targeting control siRNA (siCtrl). IL26 and CX3CL1 protein levels were markedly reduced in siRNA-transfected cells. β-Actin served as the loading control. (B) Western blot analysis of A549 and H1975 cells co-cultured with BM-CAFs transfected with siIL26 or siCtrl. IL26 knockdown in BM-CAFs significantly reduced JAK1 and STAT3 phosphorylation levels in both A549 and H1975 cancer cell lines. (C) Western blot analysis of A549 and H1975 cells co-cultured with BM-CAFs transfected with siCX3CL1 or siCtrl. CX3CL1 knockdown in BM-CAFs significantly suppressed phosphorylation levels of JAK1, STAT3, AKT, and mTOR in both cancer cell lines. (D-E) Western blot analysis of A549 and H1975 cells co-cultured with BM-CAFs transfected with siIL26, siCX3CL1, or siCtrl. Knockdown of IL26 (D) or CX3CL1 (E) in BM-CAFs significantly downregulated the expression levels of EMT and CSC markers in both cancer cell lines.


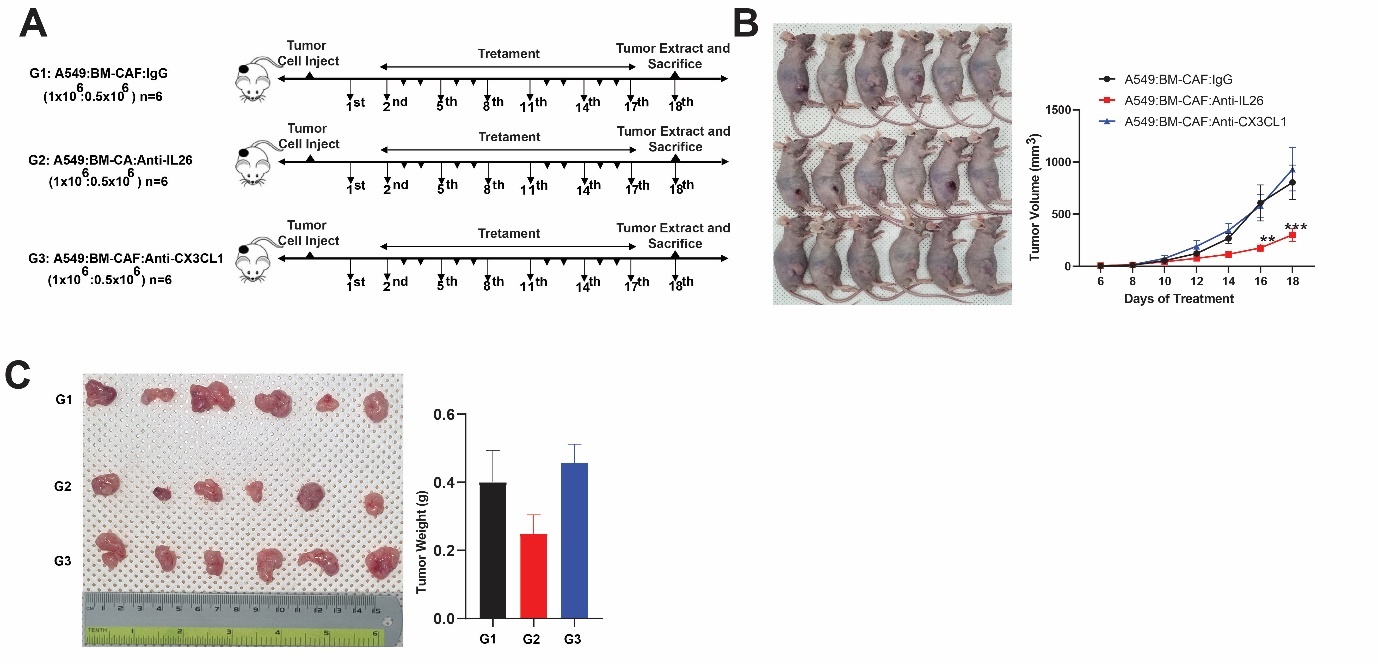


**Supplementary Figure 15.** **Neutralization of IL26 significantly reduces tumor growth in the A549 and BM-CAF co-injection xenograft model.**

(A) Schematic diagram of experimental design. Nude mice were subcutaneously co-injected with A549:BM-CAFs and divided into three treatment groups (n = 6 per group): G1 isotype control IgG, G2 anti-IL26 neutralizing antibody, G3 anti-CX3CL1 neutralizing antibody. Treatments were administered every 2 days starting from Day 2 post-injection, and mice were sacrificed on Day 18 for tumor analysis. (B) Representative images of mice from each treatment group and tumor growth curves measured over time. Tumor volume was significantly reduced in the anti-IL26 treated group compared to IgG and anti-CX3CL1 groups. Data are presented as mean ± SEM (**P < 0.01, one-way ANOVA). (C) Excised tumors and quantification of tumor weights at the endpoint. Tumors from the anti-IL26 group (G2) were visibly smaller than those from IgG (G1) and anti-CX3CL1 (G3) groups.


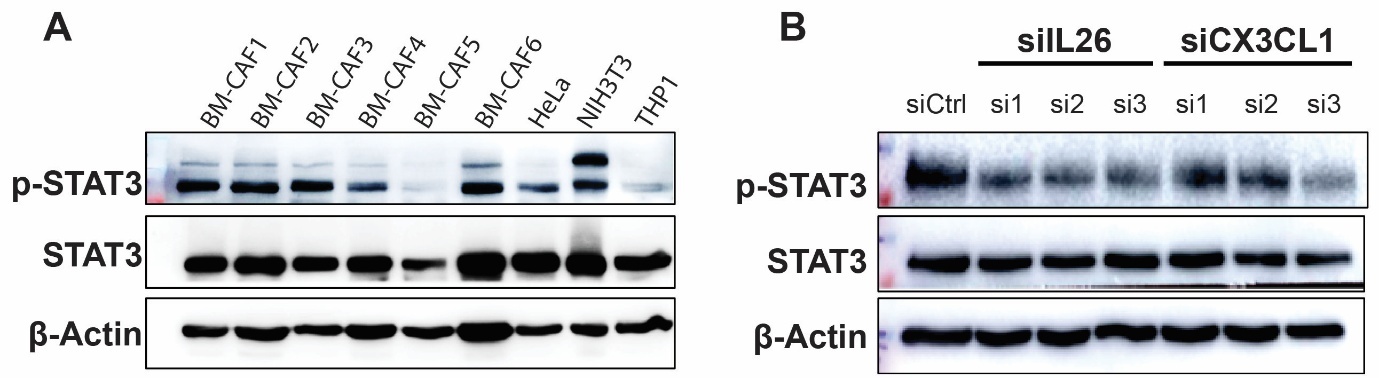


**Supplementary Figure 16.** **STAT3 signaling is consistently activated in BM-CAFs and attenuated by IL26 and CX3CL1 knockdown.**

(A) Western blot analysis of key signaling pathways in six patient-derived BM-CAFs and control cell lines (HeLa, NIH3T3, and THP-1). p-STAT3 levels were consistently elevated across all BM-CAFs, indicating activation of the STAT3 signaling in the BM-CAFs. (B) Knockdown of IL26 or CX3CL1 in BM-CAFs using three independent siRNA sequences (si1–si3) reduced STAT3 phosphorylation, implying their role in STAT3 pathway activation in BM-CAFs.


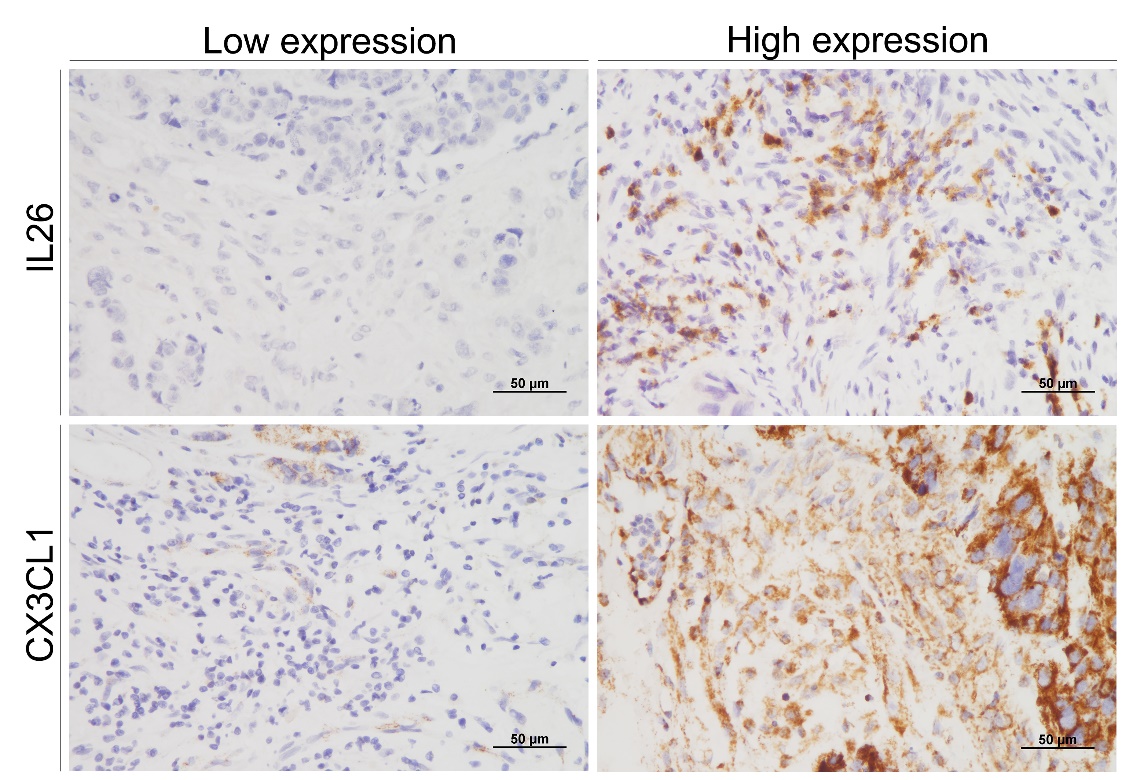


**Supplementary Figure 17.** **Immunohistochemical staining of IL26 and CX3CL1 in surgically resected BM specimens of NSCLC patients.**

Representative images show cases with low expression (top left) and high expression (top right) of IL26, and cases with low expression (bottom left) and high expression (bottom right) of CX3CL1 (original magnification, ×400). IL26 demonstrated complete absence of immunoreactivity in tumor cells, with positive staining observed only in some BM-CAFs and immune cells. In contrast, CX3CL1 showed positive immunoreactivity in BM-CAFs and immune cells, as well as in the majority of tumor cells.

**
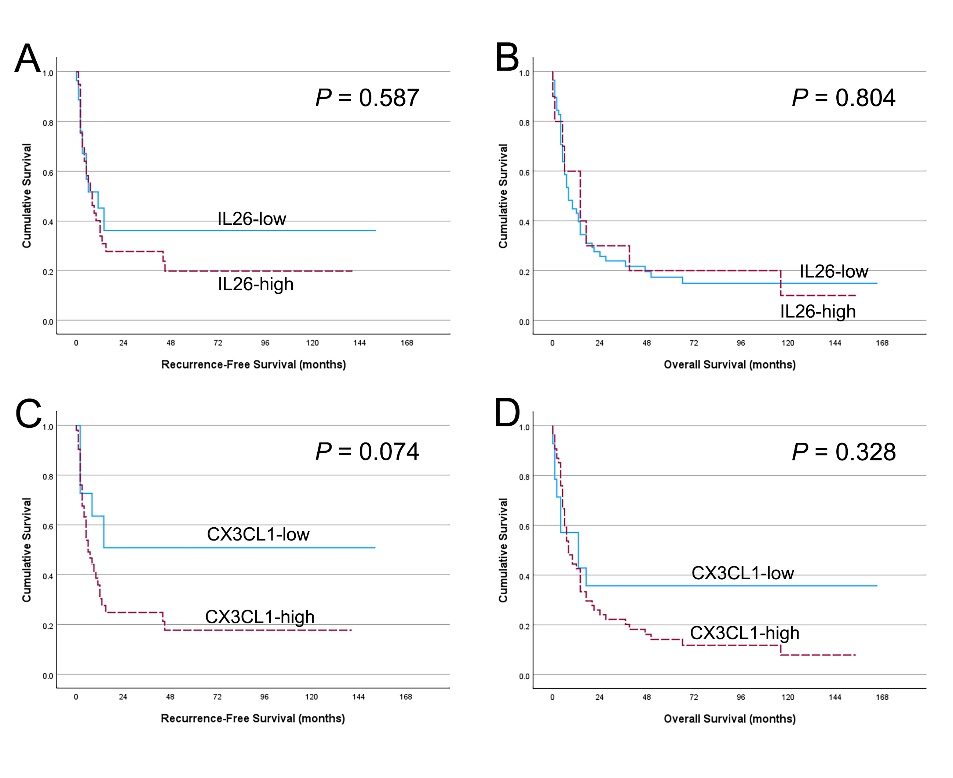
**

**Supplementary Figure 18. Kaplan–Meier survival curves in our in-house patients with BM from NSCLC according to immunohistochemical expression levels of IL26 and CX3CL1.**

(A-B) Comparison of recurrence-free survival (A) and overall survival (B) according to IL26 expression levels. Patients with high IL26 expression showed a trend toward shorter recurrence-free survival, but this difference was not statistically significant. (C-D) Comparison of recurrence-free survival (C) and overall survival (D) according to CX3CL1 expression levels. Patients with high CX3CL1 expression demonstrated a trend toward shorter recurrence-free survival with marginal statistical significance (*P*=0.074).

**Supplementary Tables**

**Supplementary Table 1. Demographic and clinical characteristics of patients-derived BM-CAF used for *in vitro & in vivo* experiments.**

| **No.** | **CAFs** | **NFs** | **Age (yrs)** | **Sex** | **NSCLC type** | **Syn/Meta** | **Location** | **Multiple/Single** | **Max. size (Cm)** | **Survival (mos, from BM resection)** |
| --- | --- | --- | --- | --- | --- | --- | --- | --- | --- | --- |
| 1 | BM-CAF1 | NF1 | 69 | M | ADC | Syn | Lt. Fr | Single | 5.4 | Death 11 |
| 2 | BM-CAF5 | NF5 | 51 | M | ADC | Syn | Lt. Fr | Single | 3 | Survive 35 |
| 3 | BM-CAF7 | NF7 | 72 | F | ADC | Meta | RT. P | Multiple | 3.8 | Death 3 |
| 4 | BM-CAF8 | NF8 | 45 | F | ADC | Syn | Rt. P | Single | 5.3 | Survive 28 |
| 5 | BM-CAF10 | NF10 | 54 | F | ADC | Meta | Rt. O | Single | 3.6 | Survive 25 |
| 6 | BM-CAF11 | NF11 | 73 | F | SQC | Meta | Rt. Cbll | Multiple | 3.4 | Death 6 |
| 7 | BM-CAF12 | NF12 | 75 | M | ADC | Syn | Lt. Cbll | Multiple | 4.7 | Survive 36 |
| 8 | BM-CAF13 | NF13 | 54 | F | ADC | Syn | Rt. Fr | Multiple | 4.6 | Survive 11 |
| 9 | BM-CAF14 | NF14 | 65 | M | NEC | Syn | Lt. Fr | Multiple | 6.3 | Survive 10 |
| 10 | BM-CAF15 | NF15 | 63 | F | ADC | Meta | Rt. Fr | Multiple | 5.3 | Survive 8 |

ADC; adenocarcinoma, BMCAF; brain metastasis CAF, CAF; cancer-associated fibroblast, Cbll; cerebellum, F; female, Fr; frontal, M; male, Lt.; left, Meta; metachronous, Max.; maximal, mos; months, NEC; neuroendocrine carcinoma, NF; normal fibroblast, NSCLC; non-small cell lung cancer, O; occipital, P; parietal, Rt.; right, SQC; squamous cell carcinoma, Syn; synchronous, yrs; years

**Supplementary Table 2. Demographic and clinical characteristics of patients-derived BM-CAF used for RNA sequence.**

| **No.** | **CAFs** | **NFs** | **Age (yrs)** | **Sex** | **NSCLC type** | **Syn/Meta** | **Location** | **Multiple/Single** | **Max. size (Cm)** | **Survival (mos, from BM resection)** |
| --- | --- | --- | --- | --- | --- | --- | --- | --- | --- | --- |
| 1 | CAF1 | NF1 | 55 | M | ADC | Syn | Lt. P | Single | 5.5 | Death 3 |
| 2 | CAF2 | NF2 | 59 | M | SQC | Meta | Rt. O | Single | 5 | Death 7 |
| 3 | CAF3 | NF3 | 61 | M | ADC | Syn | LT. P | Multiple | 4.7 | Death 3 |
| 4 | CAF4 | NF4 | 65 | M | SQC | Meta | Lt. P | Single | 5.4 | Death 9 |
| 5 | CAF5 | NF5 | 67 | M | NEC | Meta | Rt. F | Single | 5.4 | Death 12 |
| 6 | CAF6 | NF6 | 65 | M | SQC | Syn | Lt. F | Single | 4 | Death 49 |
| 7 | CAF7 | NF7 | 64 | M | SQC | Meta | Lt. F | Single | 6.8 | Death 3 |
| 8 | CAF8 | NF8 | 81 | M | SQC | Meta | Rt. O | Single | 3.7 | Death 11 |
| 9 | CAF9 | NF9 | 51 | M | NEC | Syn | Rt. Cbll | Multiple | 4.5 | Death 33 |
| 10 | CAF10 | NF10 | 75 | M | SQC | Syn | Lt. P | Single | 4 | Death 14 |
| 11 | CAF11 | NF11 | 44 | F | ADC | Meta | Rt. P | Single | 3.3 | Death 5 |
| 12 | CAF12 | NF12 | 65 | F | SQC | Meta | Lt. T | Multiple | 5 | Death 22 |
| 13 | CAF13 | NF13 | 59 | M | ADC | Syn | Lt. P | Single | 3.6 | Survive 53 |
| 14 | CAF14 | QC fail | 63 | M | ADC | Meta | Rt. F | Multiple | 6.1 | Death 4 |

ADC; adenocarcinoma, BMCAF; brain metastasis CAF, CAF; cancer-associated fibroblast, Cbll; cerebellum, F; female, Fr; frontal, M; male, Lt.; left, Meta; metachronous, Max.; maximal, mos; months, NEC; neuroendocrine carcinoma, NF; normal fibroblast, NSCLC; non-small cell lung cancer, O; occipital, P; parietal, QC; quality control, Rt.; right, SQC; squamous cell carcinoma, Syn; synchronous, yrs; years

**Supplementary Table 4. Antibodies used**

| Primary antibodies | Manufacturer | Catalog No. | Dilution |  |
| --- | --- | --- | --- | --- |
|  |  |  | IF | WB |
| ZEB1 | Bethyl | IHC-00419 |  | 1:500 |
| α-SMA | Abcam | ab7817 | 1:200 | 1:1000 |
| PDGFR-β | Thermo Scientific | MA5-15143 | 1:100 | 1:1000 |
| pan-Keratin | Cell signaling | 4545 | 1:300 | 1:1000 |
| PECAM-1/anti-CD31 | Santa Cruz | Sc-376764, |  | 1:1000 |
| SNAI1 | Santa Cruz | Sc-28199 |  | 1:1000 |
| SLUG | Abcam | Ab38551 |  | 1:500 |
| TWIST | Abcam | AB50887 |  | 1:1000 |
| Vimentin | Abcam | ab8978 |  | 1:1000 |
| CD44 | Thermo | MA4405 |  | 1:500 |
| CD133 | Ab Frontier | LF-PA50121 |  | 1:500 |
| PARP | Cell signaling | 9542 |  | 1:1000 |
| Cleaved PARP1 | Abcam | ab32064 |  | 1:1000 |
| Caspase-3 | Cell signaling | 9662 |  | 1:1000 |
| Cleaved Caspase-3 | Cell signaling | 9664 |  | 1:1000 |
| IL10Rβ | Santa Cruz | Sc-514822 |  | 1:1000 |
| IL20Rα | Santa Cruz | Sc-80065 |  | 1:1000 |
| CX3CR1 | Thermo Scientific | 702321 |  | 1:1000 |
| JAK1 | Santa Cruz | Sc-1677 |  | 1:1000 |
| phospho-JAK1 | Thermo Scientific | 44-422G |  | 1:1000 |
| AKT | Cell signaling | 9272 |  | 1:1000 |
| phospho-AKT | Cell signaling | 9271 |  | 1:1000 |
| mTOR | Thermo Scientific | AHO1232 |  | 1:1000 |
| phospho-mTOR | Thermo Scientific | 44-1125G |  | 1:1000 |
| STAT3 | Cell signaling | 30835 |  | 1:1000 |
| phospho-STAT3 | Cell signaling | 9145 |  | 1:1000 |
| Ki-67 | Abcam | ab16667 | 1:100 |  |
| α-Tubulin | Cell signaling | 3873S |  | 1:5000 |
| β-Actin | Cell signaling | 3700 |  | 1:1000 |

All secondary HRP-linked antibodies for Western Blot detection were purchased from Cell Signaling Technology. All secondary antibodies for immunofluorescence were Alexa Fluor 488/568 purchased from Invitrogen (1:500, Cat No. A11001/A11077, Thermo Fisher). All antibodies were diluted and used according manufacturer’s recommendations.

**Supplementary Table 5. Primers**

| Human genes | Primers sequence (5'-3') | Size (bp) |  |
| --- | --- | --- | --- |
| *IL26* | AGG TGT GGG TTG CTG TTA GT  GCG GTC TTC TGG AAT CGT TG | 159 |  |
| *CX3CL1* | GGC AAA CGC GCA ATC ATC TT  ATC TGC TTC TCG AAG GTG CC | 146 |  |
| *CD4* | ACC GGG GAG TCC CTT TTA GG  GGA GCC CTG ATT TCC CAG AA | 197 |  |
| *TGFB2* | | CAG CTT GTG CTC CAG ACA GT  GCT CAA TCC GTT GTT CAG GC | 200 |
| *BMP4* | | TCA CCG TTT TCT CGA CTC CG  ACG ACC ATC AGC ATT CGG TT | 193 |
| *CXCL8* | | ATG ACT TCC AAG CTG GCC GTG GCT  TCT CAG CCC TCT TCA AAA ACT TCT C | 292 |
| *ZEB1* | ATG ACA CAG GAA AGG AAG G  AGC AGT GTC TTG TTG TAG | 158 |  |
| *SNAI1* | TCG GAA GCC TAA CTA CAG CGA  AGA TGA GCA TTG GCA GCG AG | 140 |  |
| *SLUG* | CGA ACT GGA CAC ACA TAC AGT G  CTG AGG ATC TCT GGT TGT GGT | 87 |  |
| *TWIST-1* | GTC CGC AGT CTT ACG AGG AG  GCT TGA GGG TCT GAA TCT TGC T | 156 |  |
| *Vimentin* | GAC GCC ATC AAC ACC GAG TT  CTT TGT CGT TGG TTA GCT GGT | 238 |  |
| *CD133* | GGA CCC ATT GGC ATT CTC  CAG GAC ACA GCA TAG AAT AAT C | 171 |  |
| *CD44* | TGA ATA TAA CCT GCC GCT TTG  TCC GTC CGA GAG ATG CTG TAG | 73 |  |
| *GAPDH* | AGT TGT CAT GGA TGA CCT TGG C  ATC ACC ATC TTC CAG GAG CGA | 283 |  |
